# Verbal and non-verbal skills in early childhood: dimensionality, developmental trajectories, and gender differences

**DOI:** 10.3389/fpsyg.2024.1330334

**Published:** 2024-04-19

**Authors:** Magdalena Elnes, Joakim Evensen Hansen, Arne Lervåg, Ove Edvard Hatlevik, Elin Kirsti Lie Reikerås

**Affiliations:** ^1^Department of Primary and Secondary Teacher Education, Oslo Metropolitan University, Oslo, Norway; ^2^Norwegian Centre for Reading Education and Research, University of Stavanger, Stavanger, Norway; ^3^Department of Education, University of Oslo, Oslo, Norway; ^4^CREATE – Center for Research on Equality in Education, University of Oslo, Oslo, Norway; ^5^Department of Early Childhood Education, University of Stavanger, Stavanger, Norway; ^6^FILIORUM-Centre for Research in Early Childhood Education, Stavanger, Norway

**Keywords:** verbal, non-verbal, cognitive development, gender/sex differences, cognitive assessment, toddlers, preschoolers

## Abstract

This study examines the dimensionality of and relationships between two subscales from the British Ability Scales – Third Edition, measuring verbal (expressive vocabulary) and non-verbal (reasoning) cognitive skills for toddlers (age three) and preschoolers (age five), in a Norwegian context across genders. Descriptive statistics revealed item selection criteria that included specific items within each subscale. Subsequently, Confirmatory Factor Analysis established the subscales’ dimensionality (Naming Vocabulary and Picture Similarities; *N* = 1094) and confirmed measurement invariance across genders. Further, the relationships between the verbal and non-verbal factors were investigated using correlation analysis and Structural Equation Modeling. The findings revealed that the verbal factor at age three strongly predicted the verbal factor at age five and significantly influenced the non-verbal factor at age five. The non-verbal factor at age three exhibited a moderate predictive relationship with the non-verbal factor at age five, and did not significantly predict the verbal factor at age five. In terms of gender differences, girls showed higher scores on the verbal factor at age three, and a stronger correlation between the non-verbal factor at age three and the verbal factor at age five. In summary, this research provides valuable insights into cognitive skill measurement and development in a Norwegian context and highlights possible variations across gender. The study’s findings, limitations, and implications are discussed.

## 1 Introduction

Understanding early verbal and non-verbal cognitive development is crucial for unraveling the complexities of human learning and achievement. Cognitive ability measurement provides some of the best predictors of academic achievement (Johnson et al., 2006; Tikhomirova et al., 2020), occupational attainment and job performance (Gottfredson, 1997; Van Iddekinge et al., 2018), in addition to physical and mental health (Calvin et al., 2017; Lövdén et al., 2020; Peng and Kievit, 2020), despite the criticism [see Richardson and Norgate (2015)]. For example, early vocabulary skills are robust in predicting language development, including literacy, reading, academic skills, and achievement in the following years (e.g., Scarborough, 2009; Rowe et al., 2012; Dolean et al., 2021), indicating a positive influence of early verbal abilities on later development (Cunningham and Stanovich, 1997; Coyne and Harn, 2006). Non-verbal cognitive skills, such as working memory and reasoning, were found to predict school performance in primary school children (Demetriou et al., 2020). Similarly, pattern understanding, described as a non-verbal ability to detect structures and sequences of colors objects, letters, or numbers (Burgoyne et al., 2019, p. 69), was identified as a predictor of later reading and arithmetic skills (Burgoyne et al., 2019).

Despite extensive research on cognitive assessments (e.g., Kotz et al., 2008; Canivez et al., 2020), the psychometric properties of verbal and non-verbal measurements have often been insufficiently explored, particularly across gender in the Norwegian context. Investigating the consistency of cognitive assessments across genders is essential in identifying eventual measurement bias and allows for addressing potential inequalities. In Norway, a country known for its commitment to gender equality across various domains including early education and care settings, a substantial gender gap in literacy emerges at age 10 in favor of girls (Borgonovi et al., 2018). As showed in PISA 2022 results, Norway ranks sixth globally in terms of gender differences in the domain of language (OECD, 2023). Consequently, it becomes especially significant to explore early cognitive skill development in the Norwegian context, including the relationship between verbal and non-verbal cognitive skills, while considering possible gender differences in these associations.

The main aim of this study is to examine the dimensionality of two subscales, which represent verbal (expressive vocabulary) and non-verbal (reasoning) cognitive skills derived from the British Ability Scales – Third Edition (BAS 3; Elliot and Smith, 2011) within a Norwegian context across genders, and explore the relationships between the verbal and non-verbal skills measured at two – time points (age 3 and 5). By the longitudinal design and the utilization of Structural Equation Modeling (SEM) and multiple-sample Confirmatory Factor Analysis (CFA) method, we can address these issues. As a result, we can contribute to the ongoing dialog on the validity of cognitive assessments, developmental patterns in verbal (expressive vocabulary), non-verbal (reasoning) cognitive skills, and gender differences.

### 1.1 Cognitive development

Piaget’s theory of cognitive development and subsequent research have emphasized the importance of cognitive skills in children’s intellectual growth (Babakr et al., 2019). Today, psychometric cognitive test batteries, or intelligence tests, are frequently used to measure cognitive skills. These batteries consist of subtests assessing different cognitive domains, including verbal (i.e., expressive language, reading) and non-verbal skills (i.e., reasoning, spatial skills). Although these can be considered independent functions, the term general intelligence or cognitive ability, referred to as “*g*,” is widely recognized by psychologists as a higher-order factor in psychometric testing (Deary et al., 2010; Canivez and McGill, 2016; Bryan and Mayer, 2020). A modular view of intelligence remains controversial, but the effect of genetic factors on cognitive development in early childhood is considered modular, meaning that different genetic components affect specific cognitive domains. This effect becomes “molar” by influencing general cognitive functioning as the child grows into adulthood (Price et al., 2000, p. 956). Despite this, the relationship between verbal and non-verbal cognitive skills among infants is mediated by shared environmental factors rather than genetic influence (Price et al., 2000). Childhood and adult studies can produce varying results regarding the relationship between verbal and non-verbal cognitive skills, in addition to genetic and environmental influence (Price et al., 2000).

Noteworthy, adolescents and young adults with greater vocabulary skills show more rapid gains in fluid reasoning and vice versa, an effect referred to as mutualistic (Peng and Kievit, 2020). Such findings challenge the idea that cognitive functioning is purely domain-specific or entirely influenced by a single underlying “g” factor, suggesting that interrelationships between cognitive skills should be considered. For example, the Differential Ability Scales (DAS) structure organizes cognitive functioning into a hierarchy of clusters representing distinct cognitive skills (Elliott, 2001; Gordon and Elliott, 2001; Canivez et al., 2020). These clusters appear interrelated but become more distinct when the child develops. In preschool aged children, cognitive skills are clustered into verbal and non-verbal factors but become more differentiated among school-aged children, with a third cluster reflecting fluid reasoning in addition to verbal and spatial skills (Gordon and Elliott, 2001). Little is known about the development of verbal and non-verbal cognitive skill interrelations from toddlerhood to preschool age, especially regarding possible gender differences.

### 1.2 Gender/sex differences

Research on mean differences in general cognitive ability across genders has shown inconsistent results, which may relate to the use of different cognitive measures, various operationalization methods (i.e., composite scores and latent variables), and different age groups studied (Palejwala and Fine, 2015; Giofrè et al., 2022). Among toddlers and preschool children, gender differences in cognition may emerge. In one study, girls showed an advantage in general intelligence (Palejwala and Fine, 2015), although another study, including children under age 5, found no statistically significant effects of gender (Sellers et al., 2002). As mentioned earlier, general cognitive ability can be considered a multifaceted construct, reflected by the number of subtests across the various cognitive batteries. When the scores of different subtests are aggregated, these differences may offset each other (Johnson and Bouchard, 2007). For example, girls tend to outperform boys in verbal tasks from an early age (Hirnstein et al., 2023) and may demonstrate greater processing speed compared to boys (Palejwala and Fine, 2015). It is important to mention that boys tend to show greater variability in their scoring (Dykiert et al., 2009; Giofrè et al., 2022). For example, boys excel in subtests relating to visuospatial abilities, which include mental rotation tasks, spatial perception, and spatial visualization (Reilly et al., 2017), but show greater variability in mathematical and spatial abilities (Feingold, 1994). Researchers have managed to explain these differences from various standpoints, including biological theories regarding neuroanatomical differences and brain development relating to lateralization, cortical volume, and hippocampal differences (Deary et al., 2010), and socialization theories proposing that differences result from social, cultural, and other environmental factors (Wood and Eagly, 2012). Generally, an interaction of biological and environmental factors is the most likely explanation for the existence of gender differences in cognition, but has not yet been fully understood (Jäncke, 2018).

Regarding the terminology of “gender” and “sex,” sex differences refer to predominantly biological differences between males and females in chromosomes, organs, and hormones and has been used in medical or health research. Gender refers to socially constructed roles and behaviors, which can be influenced by historical and cultural factors, used as a prominent concept within the social sciences (Johnson et al., 2009; American Psychological Association, 2019; Holzhauer et al., 2020). Although we consider both terms relevant to the topic of cognitive development, we chose to use the term “gender” throughout the current paper due to its’ relevance in the field of psychology and educational sciences.

### 1.3 British ability scales

The British Ability Scales (BAS) is a battery of individual tests of distinct cognitive abilities and educational achievement and assumes a hierarchical organization of cognitive ability (Elliott, 2001). The development of BAS started in 1979 with the first version of the scale, continued by BAS-R (Elliot, 1983), the Differential Ability Scales (DAS, US adaptation of the scale; Elliot, 1990a,b), in addition to BAS II in 1996 and DAS II in 2006, before the most recent version BAS 3 was introduced (Elliot and Smith, 2011). The first version of BAS was standardized on 3,435 children, whereas the BAS II was standardized in 1995 on a smaller UK sample, including 1,689 children. Construct validity and high test–retest reliability were demonstrated (Elliott et al., 1997). Test fairness, or the degree to which the measure was equally valid for individuals from various demographic groups, including gender, was also investigated. This included a review conducted by two users sensitized to ethnic bias, psychometric assessment of item characteristics, and the prediction of educational outcomes across groups (Hill, 2005). The results indicated bias against children from minority backgrounds with a limited experience of Western culture (Elliott et al., 1997). Minimal bias effects of gender were reported, including comparable performance across ethnic groups, indicating test fairness (Hill, 2005).

The DAS II was standardized and normed in the year 2005 on a sample of 3,480 children, aged 2 years and 6 months through 17 years and 11 months, divided into 18 age groups. Each age group consisted of around 200 children, with an equal number of girls and boys in each group. The standardization included children with mild perceptual, speech, and motor impairments (Dumont et al., 2009). The reliability of DAS II was within the acceptable range between 0.87 and 0.96 for the various age groups and clusters investigated (Dumont et al., 2009). The validity studies of DAS II, including both clinical and non-clinical populations, indicated a satisfactory concurrent validity of the measure, ranging from moderate to high, as indicated by the correlation coefficients to other measures of intelligence ranging between *r = 0*.59 and *r =* 0.88 (Dumont et al., 2009). The structural validity of the DAS II was investigated using the standardization sample and both higher-order and bi-factor models indicated that the *g* factor accounted for large portions of total and common variance (Canivez et al., 2020). However, more is needed to know about the validity and test fairness of the latest version, BAS 3.

### 1.4 The current study

Based on the research gap regarding the psychometric properties of cognitive measures and cognitive developmental patterns across gender, particularly in the Norwegian context, the main aim of the current study is to investigate the dimensionality of two subscales from BAS 3 and to examine the relationships between verbal and non-verbal cognitive skills at age three and five skills, through a longitudinal design. By examining the dimensionality and stability of cognitive measures in a Norwegian context, we contribute to the literature on verbal and non-verbal cognitive skill assessment in young children. By exploring potential gender differences, we can provide additional insights regarding the existing and future assessments and the understanding of cognitive development early in life. Identifying areas in which boys and girls may excel or face challenges can guide the development of targeted interventions and educational approaches that cater to the diverse cognitive needs of each gender. Lastly, by conducting the study in a Norwegian context, we contribute to fostering inclusive educational environments and promoting equal opportunities for all children within a progressive and gender-equitable society.

Consequently, we aim to answer the following research questions:

- To what extent is the dimensionality of the verbal (Naming Vocabulary, BAS 3) and non-verbal skill assessments (Picture Similarities, BAS 3) supported at two time points in the Norwegian context? (RQ1 – Dimensionality).
- To what extent is the verbal and non-verbal cognitive skill assessments (BAS 3) invariant across genders? (RQ2 – Invariance).
- What characterizes the relationship between verbal and non-verbal cognitive skills, and what are the gender differences? (RQ3 – Relationships).
- How do verbal and non-verbal skills in toddlerhood (T1) predict subsequent preschool age (T2) skills, and what are the gender differences in these effects? (RQ4 – Prediction).

In the next sections, we present the methodology employed in the study, including the data collection, participants, and procedures, followed by the results, discussion, and conclusion.

## 2 Methods

### 2.1 Sample and procedure

The data used in the current study come from two time points (T1 and T2) of a longitudinal research project (GoBaN). The project aims to explore the quality of early childhood education and care (ECEC) centers in Norway and its possible significance for children’s development. The study was approved by the Norwegian Centre for Research Data (NSD), the Norwegian Protection Authority and conducted in compliance with GDPR, the EU, and the Research Council’s ethical standards and regulations for research. All data were stored in a secure platform for sensitive data in compliance with the Norwegian privacy regulation.

The data collection consisted of a two-step procedure. First, over 90 private and public ECEC centers were randomly drawn from seven municipalities, which were considered representative of the Norwegian population. Second, parents of children born in 2011 or 2012 who attended the selected centers were invited to participate in the study. As reported by Eliassen et al. (2017), approximately 60–70% of the parents invited to the project accepted and signed the informed written consent on behalf of their children.

The assessments were conducted in the children’s ECEC centers by trained data collectors at T1 (age three) and T2 (age five). At T1, the mean age was 2.96 years (*SD* = 0.21), and 5.02 years (*SD* = 0.12) at T2. The children were accompanied by a carrier with whom they were familiar. The accompanying carrier was informed about the test situation and that they could provide emotional but no conceptual support (Eliassen et al., 2017). The total sample comprised 1,166 children, with 543 girls (48%) and 583 boys (40 unregistered). The analytic sample comprised 1,094 children (526 girls, 564 boys, four missing), including both verbal and non-verbal factors from T1 and T2. For the verbal measure at T1, there were 7.5% missing (*N* = 1,078) and 11.7% missing (*N* = 1,029) at T2. The non-verbal measure had 6% missing at T1 (*N* = 1,096) and 11.8% (*N* = 1,028) at T2 (see Table 1). The main reason for the higher number of missing children at T2 was families relocating and changing the ECEC center (Hansen and Broekhuizen, 2021).

**Table 1:** Descriptive statistics for the total sample (*N* = 1,166) and gender groups.

Verbal	*N*	Missing (%)	Mean	*SD*	SE mean	Skewness	Kurtosis
T1	1,078	88 (7.5%)	0.55	0.24	0.007	−0.28	−0.63
Girls	516	27 (5.2%)	0.57	0.23	0.01	−0.36	−0.47
Boys	558	25 (4.5%)	0.53	0.24	0.01	−0.18	−0.74
T2	1,029	137 (11.7%)	0.43	0.18	0.006	0.06	−0.26
Girls	480	63 (13%)	0.43	0.17	0.007	0.12	−0.10
Boys	530	53 (10%)	0.44	0.19	0.008	0.01	−0.40

Non-verbal	*N*	Missing (%)	Mean	*SD*	SE Mean	Skewness	Kurtosis
T1	1,096	70 (6%)	0.70	0.18	0.005	−1.16	1.99
Girls	527	16 (3%)	0.71	0.18	0.008	−1.22	2.21
Boys	565	18 (3.2%)	0.69	0.18	0.007	−1.11	1.82
T2	1,028	138 (11.8%)	0.59	0.17	0.005	−0.71	0.53
Girls	479	64 (13.3%)	0.60	0.17	0.008	−0.74	0.74
Boys	530	53 (10%)	0.59	0.16	0.007	−0.70	0.46

### 2.2 Materials

#### 2.2.1 The British ability scales (version BAS 3)

The British Ability Scales are considered sophisticated, up-to-date cognitive test batteries, focusing on the most suitable items, reducing assessment time, and protecting the participating child’s self-esteem and motivation (Hill, 2005; Swinson, 2013). The current study used two translated (English–Norwegian) subscales (Naming Vocabulary and Picture Similarities) from the BAS 3 - Early Years Battery (Elliot and Smith, 2011). The measures were chosen above other alternative measures because of good or better predictive validity for longer-term outcomes and good reliability. In addition, they were time effective and easy to use for researchers.

The “Naming Vocabulary” subscale measures expressive vocabulary skills rather than the recognition or understanding of word meaning. The subscale consists of 36 items with age-specific start and stop points to provide children with test items appropriate to their level of ability (Elliot and Smith, 2011). Items 1 to 24 are applicable for children aged 36 to 54 months (T1), whereas items 11 to 36 are applicable for children aged 54 to 71 months (T2). During this assessment, children are given a picture they are asked to name it (i.e., chair and book). The “Picture Similarities” subscale measures non-verbal reasoning skills. The first 18 items (items 1–18) are applicable for children aged 36 to 54 months (T1), and items 12 to 35 for children aged 54 to 71 months (T2). The participating children are presented with a depicted object and a board with a row of four other depicted objects, thereby being asked to match the object with one of four alternative objects on the board. The objects have a shared element or concept, such as flowers, animals, and people, including abstract tasks containing shapes, forms, and colors. For both assessments at T1 and T2, one point is given for correctly given answers and zero points for incorrect answers.

#### 2.2.2 Covariates

Parents or legal guardians of the participating child provided the child’s national identification number in a questionnaire, which included information regarding gender and age. To preserve anonymity in the dataset, “age at test” was calculated by the use of birth date and the reported date of both “Naming Vocabulary” and “Picture Similarities” assessments at T1 and T2. Consequently, the exact age of the children was recorded at both time points. Age at test for T1 was used to account for the differences in age among the children in the sample.

### 2.3 Analytical approach

Descriptive statistics were derived using IBM SPSS, version 27 (IBM Corp, 2020). The remaining analyses, including correlations, Confirmatory Factor Analysis (CFA), and Structural Equation Modeling (SEM), were performed using the Mplus version 8 software (Muthén and Muthén, 1998–2011). SEM is a confirmatory approach that can be used for validation through CFA and regression with latent variables (Kline, 2016). In SEM analyses, latent variables correspond to hypothetical constructs or explanatory entities presumed to reflect concepts that cannot be directly observed, such as intelligence (Kline, 2016). Acceptable levels of factor loadings and Fitness Indexes thresholds indicate the suitability of items in measuring their respective latent constructs.

In the current study, the cognitive tests include items with categorical data. Hence, the Weighted Least Squares Mean and Variance adjusted (WLSMV) estimator was chosen as an appropriate robust estimator for categorical indicators (Muthén et al., 2015). Because the Chi-square difference testing can be sensitive to sample size, it may lead to a rejection of a satisfactory model (Chen, 2007; Kline, 2016). Consequently, the comparative fit index (CFI), the Tucker-Lewis Index (TLI), and the root mean square error of approximation (RMSEA) were used as fit indices. For the current study, we considered values TLI > 0.95, CFI > 0.95, and RMSEA <0.06 (Hu and Bentler, 1999) as indicative of adequate fit, for categorical indicators and the WLSMV method.

The proportion of missing values ranged between 6 and 13%. The data was adjusted after inspecting the distribution of the cognitive measure data. When mapping the items on the included assessment, too many (>90%) or very few (<10%) have achieved correct responses. These items can appear as outliers and can provide little information for analysis. In the current analysis, little variance resulted in empty cells in cross-tabulations, potentially leading to bias in the model. These items were thus removed. There may be uncertainty related to removing items. Hence, we compare the adjusted data findings with analyses including all items (see Supplementary Appendix D).

Despite support regarding the validity of both DAS II and BAS II, more information is needed about the validity of BAS 3, especially for a translated version in a Norwegian sample. Consequently, four confirmatory factor analysis (CFA) models were fitted on the total sample to investigate the factor structure of the non-verbal cognitive and verbal cognitive assessments measured by Picture Similarities and Naming Vocabulary of BAS 3 at two time points (age 3 – T1; age 5 – T2) in a Norwegian sample (RQ1). Scale reliability (*ω*) was calculated based on the CFA results in accordance with Raykov (2001). Further, a two-group CFA with gender as the grouping variable was implemented to test the factors across gender. Scalar measurement Invariance (MI) testing across gender was performed to explore whether the scores of the latent factors have equal meaning across groups (RQ2) and whether score differences can be attributed to group membership (Horn and McArdle, 1992). Metric invariance could not be tested due to the categorical nature of the data. Based on uncertainty relating to the chi-square testing, additional cut-off criteria for CFA (<−0.010) and RMSEA (<−0.015) change were applied, which would indicate scalar non-invariance (Chen, 2007).

**Figure 1:**
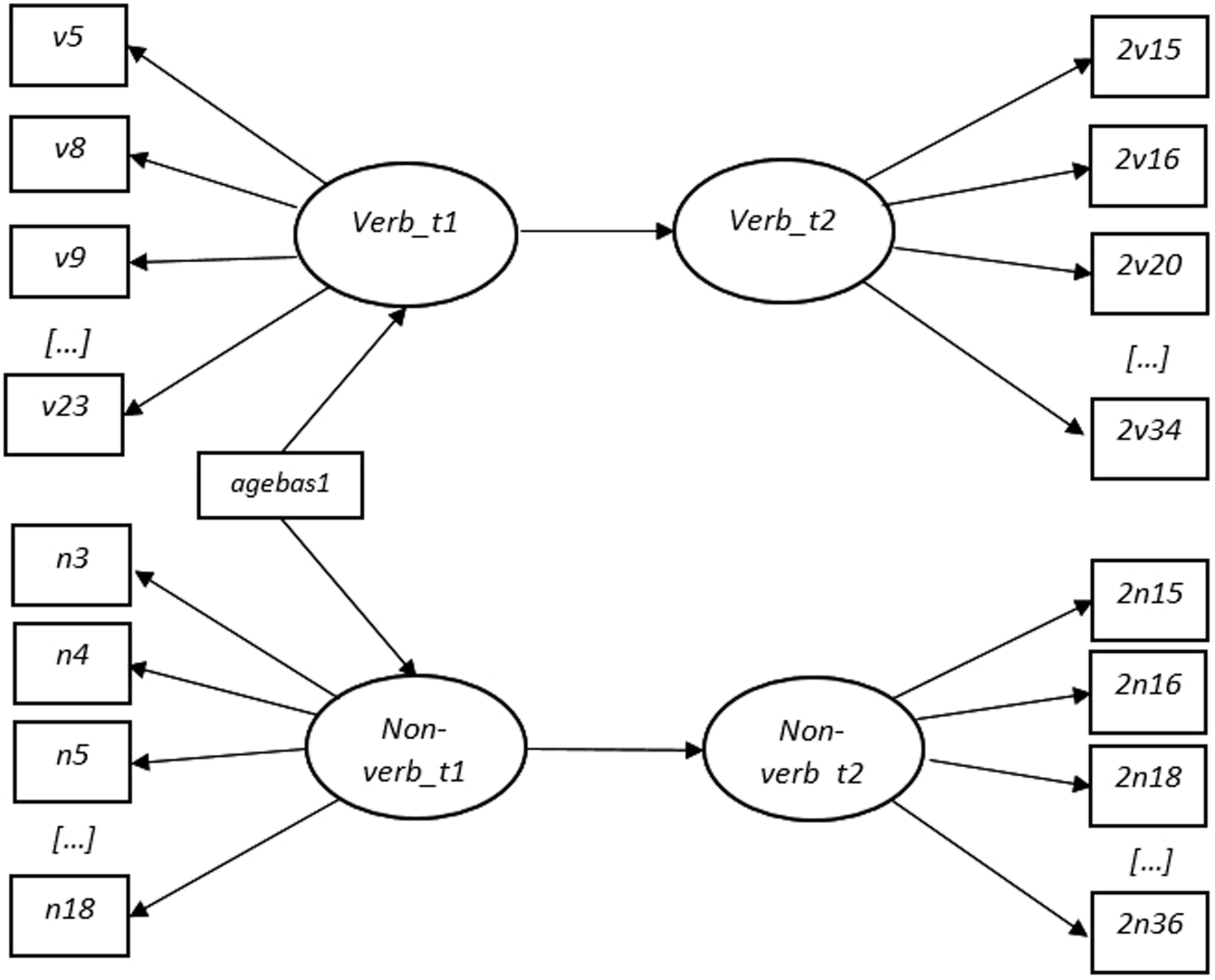
First Step of SEM Analysis with Naming Vocabulary (Verb_t1 and Verb_t2) and Picture Similarities (Non-verb_t1 and Non-verb_t2) as latent variables (oval circle) on time 1 (t1) and time 2 (t2). Verb_t1 influences Verb_t2 and Verb_t1 influences Verb_t2. Age (agebas1) is included as observed variable with prediction on Verb_t1 and Non-verb_t2.

**Figure 2:**
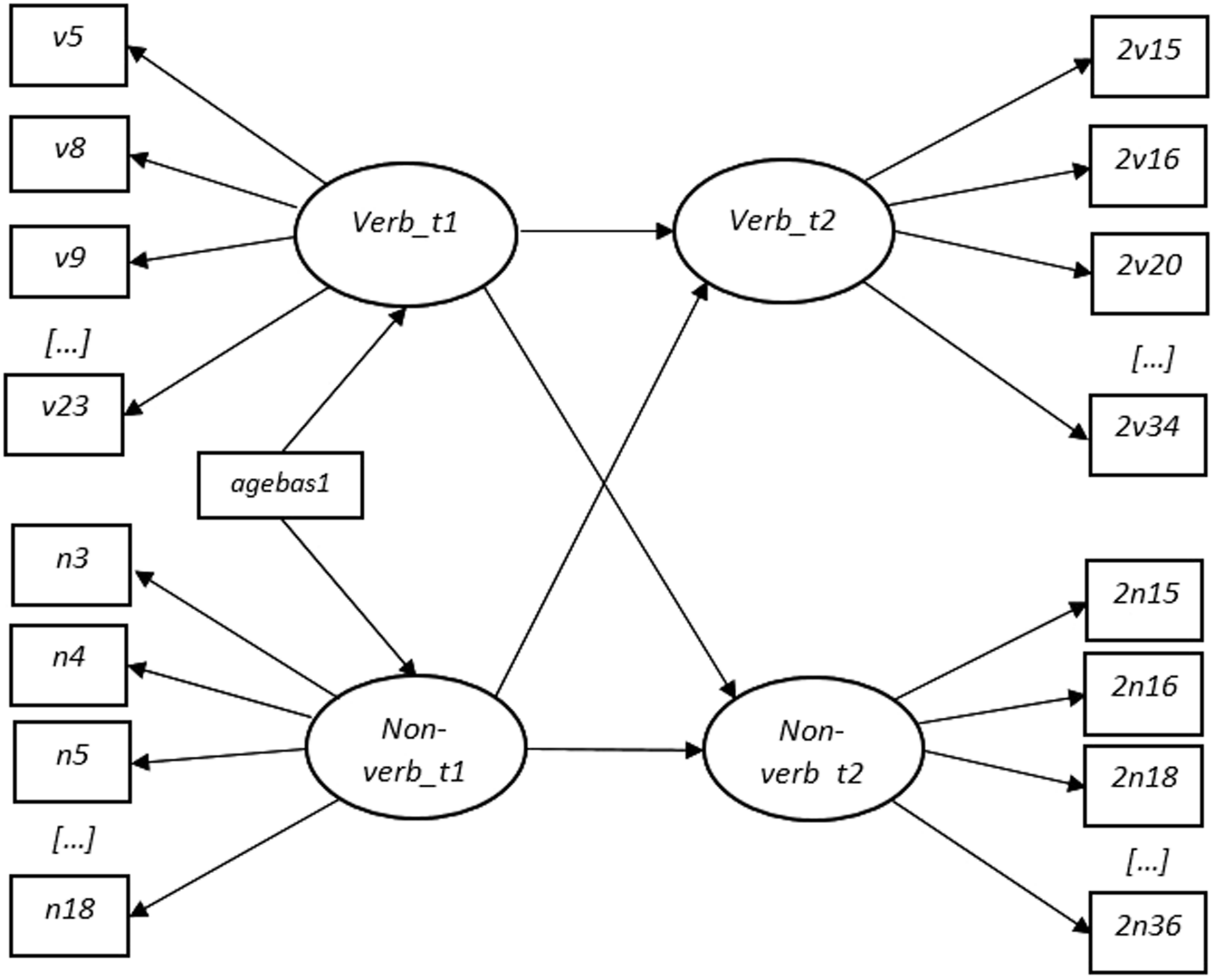
Second Step of SEM Analysis with Naming Vocabulary and Picture Similarities as latent variables. An addition to Figure 1 is that Verb_t1 influences Non-verb_t2 and Non-verb_t1 influences Verb_t2.

The relationships between the latent factors representing verbal and non-verbal skills measured at two time points were investigated through correlation analysis and compared across groups and time points using the “model test” option in Mplus (RQ3). Subsequently, structural Equation Modeling (SEM) was applied to test whether verbal and non-verbal latent factors measured at T1 could predict their respective latent factors measured at T2. In the first step (Figure 1), age at test for T1 was set as an observable variable predictive of their respective factors representing verbal and non-verbal cognitive skills. Age at test for T2 was not controlled for due to similar variation in T2 age among the participants. In the second step (Figure 2), latent variables measured at T1 were additionally set as predictors for the latent variables measured at T2 (RQ4).

## 3 Results

The current section aims to answer the overarching research question regarding cognitive skill measurement and development in a Norwegian sample. Consequently, we explore the validity of two subscales from BAS 3 measuring verbal (Naming Vocabulary) and non-verbal (Picture Similarities) cognitive skills in a Norwegian context across genders and investigate the relationships between these skills in both boys and girls. First, descriptive statistics, including raw scores, will be presented before the investigations of each research question.

### 3.1 Descriptive statistics

Items with less than 90% and more than 10% correct response percentage in the total sample from the Naming Vocabulary and Picture Similarities subscales were included in the analysis. The remaining items were deleted. Consequently, the Naming Vocabulary T1 subscale included 16 items, in which the correct response percentages ranged from 18.5 to 88.5%. At T2, the Naming Vocabulary subscale included 13 items, with correct response percentages ranging between 11 and 83.6%. The Picture Similarities subscale at T1 included 16 items, whereas T2 consisted of 20 items. For T1, the correct response percentage ranged from 33 to 89%, and from 17 to 88% for T2. All of the included items were used to derive descriptive statistics, including sample size, missing values, means, standard deviations, item range, skewness, and kurtosis values (Table 1). The distribution of the subscales and the correlation matrix of the individual items can be seen in Supplementary Appendices A,E, respectively.

### 3.2 Dimensionality and invariance (RQ1 and RQ2)

Four individual CFA models were fitted on the total sample to determine the extent to which the dimensionality of the verbal and non-verbal cognitive skill measurements (Naming Vocabulary and Picture Similarities, BAS 3) is valid across two time points and gender in a Norwegian context. Scale reliability, omega (ω), was calculated (Raykov, 2001). The results are presented for each assessment at each time point, including the results for the total sample (Table 2), item parameter estimates (Supplementary Appendix B), and measurement invariance test results with gender as the grouping variable (Table 3).

**Table 2:** CFA fit indices results for the total sample.

	χ^2^ (df)	*p*-value	CFI	TLI	RMSEA	90% CI
Verbal T1	378.121 (104)	0.000	0.964	0.959	0.049	0.044–0.055
Verbal T2	125.187 (65)	0.000	0.961	0.954	0.030	0.022–0.038
Non-verbal T1	164.350 (104)	0.000	0.956	0.950	0.023	0.016–0.030
Non-verbal T2	262.776 (170)	0.000	0.949	0.942	0.023	0.017–0.028

**Table 3:** Two-group CFA fit indices and measurement invariance test results.

	Model	Nr of *P*.	χ^2^	df	*p*-value	CFI	TLI	RMSEA
Verbal T1	Configural	64	452.184	208	0.000	0.968	0.963	0.047
	Scalar	50	464.640	222	0.000	0.968	0.965	0.045
	Comparison		23.368	14	0.055			
Verbal T2	Configural	52	183.255	130	0.002	0.964	0.957	0.028
	Scalar	41	197.241	141	0.001	0.962	0.958	0.028
	Comparison		15.737	11	0.151			
Non-verbal T1	Configural	64	258.220	208	0.010	0.963	0.957	0.021
	Scalar	50	272.826	222	0.011	0.963	0.960	0.020
	Comparison		16.582	14	0.279			
Non-verbal T2	Configural	80	402.835	340	0.011	0.961	0.956	0.019
	Scalar	62	422.425	358	0.011	0.960	0.957	0.019
	Comparison		16.927	12	0.255			

The first model, including the total sample and items from the verbal assessment at T1, showed acceptable fit indices. All standardized factor loadings were above the 0.4 threshold (Mehmetoglu and Jakobsen, 2017), ranging from 0.442 to 0.825. The scale reliability coefficient was ω = 0.936. The two-group CFA with gender as the grouping variable showed good model fit indices as well, with standardized factor loadings from 0.346 to 0.814 among girls, and from 0.461 to 0.836 among boys. Support for scalar invariance was found regarding the chi-square test, χ^2^ (14) = 23.368, *p* = 0.055, and in accordance with the CFI and RMSEA cut-off criteria (Chen, 2007). There was a significant difference in the factor mean score, where boys had an average standardized score of 0.204 lower than girls (*SD* = 0.067, *p = 0*.002), indicating a small effect size. The second model, including the verbal assessment at T2, showed a good fit with standardized factor loadings ranging from 0.229 to 0.702. Only two items showed factor loadings below the 0.4 threshold (items 12 and 26). Scale reliability was 0.838. The results of the two-group CFA were further lending support for the verbal T2 model. The standardized factor loadings ranged between 0.213 and 0.684 in the group of girls. Among boys, the factor loadings ranged between 0.229 and 0.731. We found scalar invariance across the gender groups, as indicated by χ^2^ (11) = 15.737, *p* = 0.151, and a slight change in CFI of −0.002.

The third CFA model consisted of items from the non-verbal T1 assessment, which showed acceptable results. The standardized factor loadings ranged from 0.319 to 0.692, but five items showed weak factor loadings below the 0.4 threshold. Scale reliability was at 0.819. The two group CFA model resulted in acceptable fit indices, with standardized factor loadings ranging between 0.337–0.755 among girls and between 0.310–0.636 in the group of boys. Support for scalar invariance was found, with χ^2^ (14) = 16.582, *p* = 0.279, and no changes in the CFI value (0.963). The fourth CFA model of the non-verbal T2 assessment showed acceptable results.

The standardized factor loadings ranged from 0.284 to 0.623. Most factor loadings exceeded the 0.4 threshold except for four items (items 13, 23, 33, and 35). The calculated scale reliability was 0.842. The two-group CFA model indicated a better fit, with standardized factor loadings ranging from 0.273 to 0.646 among girls, and from 0.266 to 0.629 among boys. The chi-square test of measurement invariance with χ^2^ (12) = 16.927, *p* = 0.255, and the CFI change of −0.001 indicated scalar invariance across gender.

### 3.3 Relationships between the factors (RQ3)

A correlation analysis with latent factors was conducted to investigate the nature of the relationships between verbal and non-verbal cognitive skills at ages 3 and 5 (Supplementary Appendix C, S7). The model including all four factors indicated a good fit: χ^2^ = 2435.450 (2009), *p* < 0.001. RMSEA = 0.014 (CI = 0.012–0.016), CFI = 0.965, TLI = 0.963. The correlation analysis showed that the correlation between verbal factors at T1 and T2 was *r* = 0.66 (*p* < 0.001), and *r* = 0.27 (*p* < 0.001) between the non-verbal factors at two different time points (T1 and T2). The correlation of the verbal factors was significantly stronger compared to the non-verbal factors. Further, the correlation between the two assessments representing verbal and non-verbal cognitive skills at T1 was *r* = 0.41 (*p* < 0.001) and *r* = 0.38 at T2. This difference was not statistically significant. Lastly, the correlation between the verbal factor at T1 and the non-verbal factor at T2, as well as the verbal factor at T2 and the non-verbal factor at T1, was *r* = 0.24 (*p* < 0.001).

The relationships between the factors were investigated with two-group CFA (Supplementary Appendix C, S8), which showed acceptable results: χ^2^ = 4417.130 (4075), *p* < 0.001. RMSEA = 0.012 (CI = 0.012–0.015), CFI = 0.965, TLI = 0.965. One correlation was significantly different between the gender groups according to the Wald test (Supplementary Appendix C, S9); girls showed a stronger correlation between non-verbal T1 factor and verbal T2 factor compared to boys [4.275 (1), *p* = 0.0387].

### 3.4 Prediction (RQ4)

**Figure 3:**
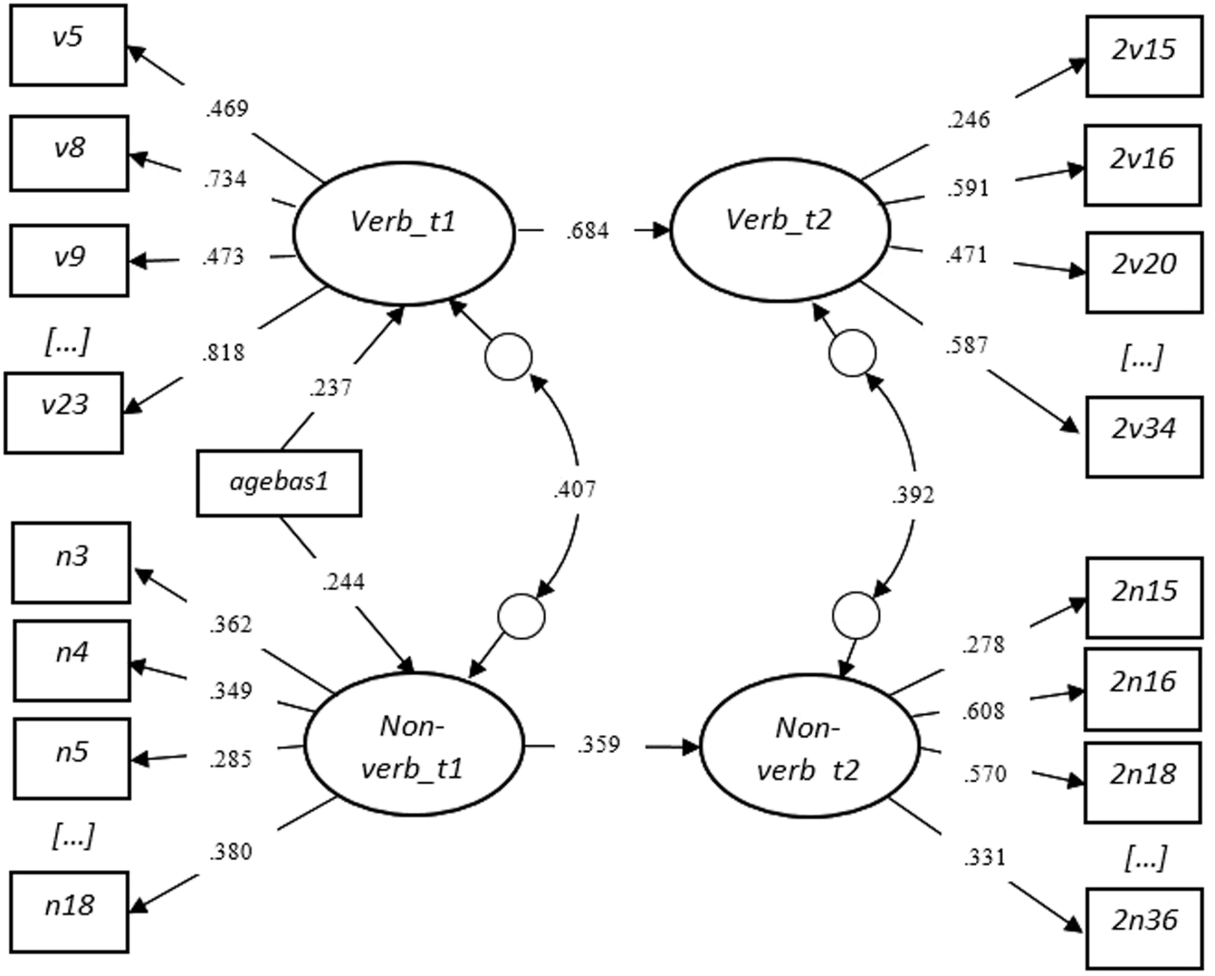
SEM Step One - Standardized Results with the Total Sample (Testing Figure 1).

To investigate the predictive value of verbal and non-verbal skills measured in toddlerhood (T1) for subsequent skills in preschool age (T2) and examine whether there are gender differences in these effects, we divided the analysis into two steps. In the first step (Figure 3), the relationship between verbal and non-verbal cognitive skills measured at T1 and T2 was tested for the total sample. Two factors measured at T1 were set as predictive of their respective factors from T2. Simultaneously, a correlation between verbal and non-verbal skills both at T1 and T2 was investigated. The overall model fit was good: χ^2^ = 2551.724 (2074), *p* < 0.001. RMSEA = 0.015 (CI = 0.012–0.016), CFI = 0.957, TLI = 0.955, WRMR = 1.099.

**Figure 4:**
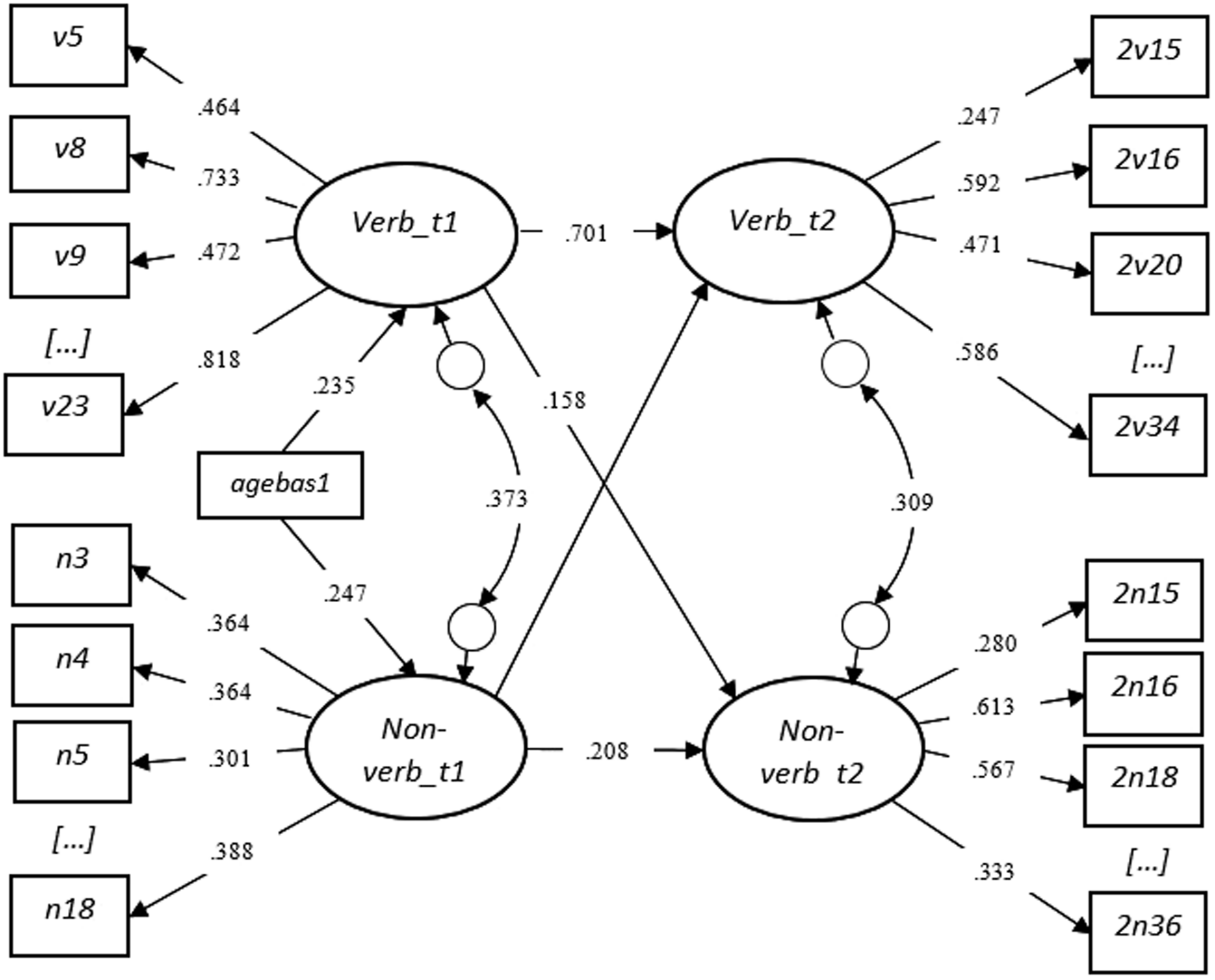
SEM Step Two - Standardized Results with the Total Sample (Testing Figure 2).

In the second step (Figure 4), the verbal factor from T1 was set as a predictor of the non-verbal T2 factor. Similarly, the non-verbal factor from T1 was set as a predictor of the verbal factor from T2. The overall model fit showed good results: χ^2^ = 2505.505 (2072), *p* < 0.001. RMSEA = 0.014 (CI = 0.012–0.016), CFI = 0.961, TLI = 0.959, WRMR = 1.078. The chi-square (χ^2^) difference test was applied with the “DIFFTEST” option in Mplus (Muthén and Muthén, 1998–2011, p. 508) to investigate whether the first model (step one) differs from the second model (step two) significantly. The results indicated that the second model is statistically a better fit (Δχ^2^ = 15.819, Δdf = 2, *p* < 0.001). Further results showed that the verbal factor at T1 remained a significant predictor of the verbal factor from T2, with a weak but significant coefficient on the non-verbal ability factor at T2 (unstandardized *b* = 0.09, *p* < 0.001). The non-verbal factor from T1 predicted the non-verbal factor at T2 significantly, but not the verbal ability factor at T2. Age at test was a significant predictor of both verbal and non-verbal cognitive factors at T1. The residual correlations of the latent factors at T1 and T2 were significant, but did not differ significantly [0.978 (1), *p* = 0.323].

Lastly, gender was included in the analysis as a grouping variable. The model fit was good χ^2^ = 4555.344 (4201), *p* < 0.001. RMSEA = 0.012 (CI = 0.009–0.015), CFI = 0.961, TLI = 0.960, WRMR = 1.424. However, none of the coefficients were significantly differing across genders, except for the non-significant coefficient of the non-verbal factor at T1 on the verbal factor at T2 [5.390 (1), *p* = 0.0203]. Noteworthy, this coefficient appeared as negative and marginally significant among boys, with a standardized beta value of *β* = −0.104, *p* = 0.069 (unstandardized *b* = −0.146, *p* = 0.078).

## 4 Discussion

In the current study, our goal was to address the dimensionality and validity of two subscales from BAS 3 measuring verbal (Naming Vocabulary) and non-verbal (Picture Similarities) cognitive skills in a Norwegian context across genders, and to investigate the nature of the relationships between verbal and non-verbal cognitive skills in toddlerhood and preschool age, across gender. Overall, the results showed that the scales are valid for both age groups and among boys and girls. However, the moderate relationship between the non-verbal assessments at T1 and T2 may indicate scale inconsistency across time. Regarding the gender differences, we found a small difference in the verbal factor in toddlerhood (T1) in favor of girls, and a stronger association between the non-verbal factor in toddlerhood and the verbal factor in preschool age (T2) among girls, indicating a subtle gender difference in cognitive developmental patterns. Before discussing the findings of our study in detail, it is essential to emphasize the importance of the outcomes, which provide valuable insights into the validity of the assessments and the complex dynamics of cognitive skills during early childhood while considering the influence of gender, thus enriching our comprehension of early cognitive skill development.

### 4.1 Dimensionality and scale applicability (RQ1)

In the current study, we found support for the dimensionality of the two BAS 3 subscales, Naming Vocabulary and Picture Similarities (RQ1) at two time-points, ages three and five. Despite most factor loadings being above the 0.4 threshold, some items indicated slight variance explained by their respective latent factors, reflecting possibly problematic measurement even after item removal. Due to the dichotomous nature of the data, the low factor loadings could result from a low variance in these items as well, with very many or very few correct responses.

Prior to item removal, most of these removed items showed a high, rather than low response rate, indicating that the scale was relatively easy for the sample studied. When mapping multiple-choice items, as in the “Picture Similarities” subscale with four alternative answers for the participating child, using items with more than 75% or less than 25% correct responses is generally recommended. However, this threshold was considered too strict for the current data, resulting in an inappropriate number of items for analysis. Another standard threshold includes a 90–10% and a 95–5% ratio of the response rate. In the current study, due to little variation in the data and empty cells in the cross-tabulations of the items that could potentially lead to bias in the analyses, we used the 90–10% threshold. It is worth noticing that this threshold removed more than 20% of the original items from the “Naming Vocabulary” subscale at both time-points. Whether the resulting latent factors are valid representations of the measured, theoretical verbal concept could be discussed.

The analyses encompassing all items revealed that we were unable to confirm measurement invariance across gender in the verbal factors at ages three and five, nor did we find support for the overall model fit of the verbal factor at age five (see Supplementary Appendix D). However, these results could be biased due to the problem with empty cells in the cross tabulations, as indicated by our results. Consequently, we recommend using the revised version of the Naming Vocabulary subscale in a Norwegian context, provided in the current study, which may additionally serve as a more effective verbal and non-verbal cognitive assessment. The overall patterns of the investigated relationships was not changed based on the item removal (Supplementary Appendix D).

It is essential to note that BAS 3 was developed in English-speaking countries; the response rate in the current sample could relate to cultural and lingual differences. For example, the included words in the Naming Vocabulary subscale may have varying difficulty and age of acquisition in English versus Norwegian language (see Ortiz and Oganes, 2022). Furthermore, the families of the studied sample showed higher socioeconomic status (SES) than the average SES in Norway (e.g., Hansen and Broekhuizen, 2021). Family SES could potentially have an indirect influence on the participating child’s cognitive abilities, as indicated in earlier studies (Pace et al., 2017; Romeo et al., 2022). The low variability caused by a high correct response percentage among the participating children could be related to higher family SES in the studied sample. Consequently, we consider the latent factors in the current analyses to consist of items representative of their theoretical concepts, and the resulting assessment as appropriate and effective for a Norwegian sample. Noteworthy, without easy or difficult items in the assessments, it can be problematic to investigate children who score low or high on verbal and non-verbal cognitive skills. In such cases, a different approach including easy and difficult items would be more suitable, but this is outside the scope of the current study.

### 4.2 Gender invariance (RQ2)

In the current study, we found support for measurement invariance at configural and scalar levels and dimensionality of the two BAS 3 subscales, Naming Vocabulary and Picture Similarities, and across genders (RQ2) in a Norwegian context, at ages three and five. Traditionally, scores from individual tests, known as observed scores, have been used to assess differences in cognitive functions. However, observed scores consist of measurement errors and unique variations that may affect the accuracy of eventual comparisons across groups, including gender. Using latent variables estimated through SEM gives a more reliable approach to measuring cognitive skills by removing sources of unreliability, providing a purer measure of the underlying construct. For example, observed and latent variable approaches may yield different results (Steinmayr et al., 2010). The importance of the latent variable approach is emphasized specifically to investigate gender differences through measurement invariance testing.

Configural invariance indicates that the latent construct structure is similar across the groups studied and that the pattern of relationships between the latent variables and indicators is consistent (Kline, 2016). Scalar invariance represents comparable measurement scales, and differences between the groups can be attributed to the latent constructs rather than being a result of differences in how the items are understood or responded to Kline (2016). Scalar invariance allows further analyses of latent mean differences, which showed that girls scored higher on average compared to boys in T1 (age three). The standardized coefficient of 0.2 in favor of girls indicated a small gender difference in magnitude. The results are comparable to a previous study performed by Hansen and Broekhuizen (2021) using a similar sample to the one in the current study. The authors investigated gender differences in BAS 3 verbal ability scores, given as an observable variable rather than a latent variable, calculated in accordance with the BAS 3 manual (Elliot, 2011; Elliot and Smith, 2011). The similarity between the results suggests that the latent factor approach reflects the original ability score, lending support for the validity of the latent verbal factors in this study. In sum, the results suggested acceptable fit indices, indicating that the proposed model representing the theoretical relationships among the variables fits the observed data adequately (Kline, 2016). Consequently, the data and the latent factors representing verbal and non-verbal skills demonstrate dimensionality and stability across gender in the Norwegian context.

### 4.3 Relationships between the factors (RQ3 and RQ4)

The relationships and effects measured between the latent factors, representing verbal and non-verbal cognitive skills measured at the age of three (T1) and five (T2), were investigated using correlation and regression analysis through SEM modeling. The results showed moderate to strong relationships between the verbal factors at T1 and T2, lending further support for the validity of the Naming Vocabulary subscale of BAS 3 (Elliot and Smith, 2011). However, the weak relationships between the non-verbal factors at T1 and T2, measured by the Picture Similarities scale of BAS 3, indicated little common variance. These findings suggest that the non-verbal factors represent two independent constructs across time. Generally, early cognitive skills may undergo significant developmental changes and become differentiated, as observed in preschool and school-aged children (Gordon and Elliott, 2001). Despite this, we did not find significant differences in the correlations between verbal and non-verbal cognitive skills in toddlerhood (T1; age three) and in preschool-aged children (T2; age five), suggesting the same level of dependency and possible mutualistic effects (Kievit et al., 2017). Although the relationship strength between verbal and non-verbal cognitive skills was similar across time, the current results demonstrate that the concept of non-verbal cognitive skills may be different in children aged three compared to children aged five.

We found significant gender differences in the correlational strength between the non-verbal factor at T1 and the verbal factor at T2, in favor of girls. Furthermore, we found a significant gender difference in the regression coefficient of the non-verbal factor (T1) on the verbal factor (T2), although the coefficient appeared non-significant, and marginally significant in the group of boys. Despite this, it is important to point out that the coefficient was negative in the group of boys. Overall, these findings suggest that during cognitive development, the impact of non-verbal skills on later verbal skills may differ across genders and show stronger associations among girls. As mentioned earlier, the relationship between verbal and non-verbal cognitive skills is complex and may vary due to changes in development as a function of age, as well as varying associations with genetic and environmental factors (Price et al., 2000).

## 5 Limitations

There are some limitations in the current validation study. Firstly, the study did not include the full BAS 3 scale, which consists of several subscales representing various aspects of cognition that can be calculated into a “g” factor. Including all of the subscales would give a more nuanced picture of early cognitive development. Secondly, we did not include covariates such as family SES, which could be relevant to investigate in the current study. The third limitation relates to measurement invariance testing across time. Due to the exclusion of a relatively large number of items, it was impossible to test for measurement invariance across T1 and T2. The possibility of doing so could have given a more appropriate answer to the question regarding the relationship between T1 and T2 factors, resulting in a satisfactory addition to the performed analyses. It is also crucial to acknowledge the non-randomized sample selection, emphasizing the need for caution when generalizing the current findings to a broader population. Finally, the translation of the Naming Vocabulary subscale lacked cultural adaptation, such as considering developmental patterns of specific words, requiring further research to support the subscale’s validity.

## 6 Conclusion and further research

In conclusion, the results indicate that the adaptation of two BAS 3 subscales using the latent variable approach, was appropriate within the Norwegian context. The Naming Vocabulary subscale appeared to be valid and invariant across genders. Girls showed higher latent mean on verbal ability factor than boys at age three, but not at age five. Furthermore, the latent factor representing verbal abilities at age three predicted verbal and non-verbal abilities at age five. The Picture Similarities subscale appeared valid in the Norwegian context and across genders as well. However, it showed more significant concerns regarding its validity due to the weak relationship across time and the non-significant predictive relationship to the verbal factor at age five. Gender differences were found in the relationship between the non-verbal factor measured at T1 and the verbal factor measured at T2, indicating a stronger relationship between the two within the group of girls.

The observed results highlight the necessity for continued research and the importance of gender differences in both means and effects, to increase the understanding of the complex dynamics in early cognitive skill development. Consequently, further research should investigate the underlying factors contributing to the observed effects and gender differences, such as various aspects of the early childhood environment and their interactions with gender. Cross-cultural comparisons can illuminate the interplay between culture, gender, and cognition, whereas neurobiological research can provide a holistic view on cognitive development. Overall, meta-analyses and replication studies can further validate and extend our findings.

## Data availability statement

The data analyzed in this study is subject to the following licenses/restrictions: data not available due to ethical/legal restrictions. Requests to access these datasets should be directed to www.goban.no (goban@oslomet.no).

## Ethics statement

The studies involving humans were approved by Norwegian Centre for Research Data (NSD) and Norwegian Protection Authority. The studies were conducted in accordance with the local legislation and institutional requirements. Written informed consent for participation in this study was provided by the participants’ legal guardians/next of kin.

## Author contributions

ME: Data curation, Formal analysis, Investigation, Methodology, Validation, Writing – original draft, Writing – review & editing, Conceptualization, Software. JH: Conceptualization, Investigation, Methodology, Supervision, Writing – review & editing. AL: Formal analysis, Methodology, Supervision, Validation, Writing – review & editing. OH: Conceptualization, Investigation, Methodology, Supervision, Visualization, Writing – review & editing. ER: Conceptualization, Methodology, Supervision, Writing – review & editing.

## References

[ref1] American Psychological Association (2019). Publication manual of the American Psychological Association. 7th Edn. Washington, DC: APA.

[ref2] Babakr Z. Mohamedamin P. Kakamad K. (2019). Piaget’s cognitive developmental theory: critical review. Educ. Q. Rev. 2, 516–524. doi: 10.31014/aior.1993.02.03.84

[ref3] Borgonovi F. Choi Á. Paccagnella M. (2018). “The evolution of gender gaps in numeracy and literacy between childhood and adulthood” in OECD education working papers, 184 (Paris: OECD Publishing)

[ref4] Bryan V. M. Mayer J. D. (2020). A meta-analysis of the correlations among broad intelligences: understanding their relations. Intelligence 81:101469. doi: 10.1016/j.intell.2020.101469

[ref5] Burgoyne K. Malone S. Lervag A. Hulme C. (2019). Pattern understanding is a predictor of early reading and arithmetic skills. Early Child. Res. Q. 49, 69–80. doi: 10.1016/j.ecresq.2019.06.006

[ref6] Calvin C. M. Batty G. D. Der G. Brett C. E. Taylor A. Pattie A.. (2017). Childhood intelligence in relation to major causes of death in 68 year follow-up: prospective population study. BMJ 357:j2708. doi: 10.1136/bmj.j2708 28659274 PMC5485432

[ref7] Canivez G. L. McGill R. J. (2016). Factor structure of the differential ability scales–second edition: exploratory and hierarchical factor analyses with the core subtests. Psychol. Assess. 28, 1475–1488. doi: 10.1037/pas0000279, PMID: 27046278

[ref8] Canivez G. L. McGill R. J. Dombrowski S. C. (2020). Factor structure of the differential ability scales–second edition Core subtests: standardization sample confirmatory factor analyses. J. Psychoeduc. Assess. 38, 791–815. doi: 10.1177/0734282920914792

[ref9] Chen F. F. (2007). Sensitivity of goodness of fit indexes to lack of measurement invariance. Struct. Equ. Model. 14, 464–504. doi: 10.1080/10705510701301834

[ref10] Coyne M. D. Harn B. A. (2006). Promoting beginning reading success through meaningful assessment of early literacy skills. Psychol. Sch. 43, 33–43. doi: 10.1002/pits.20127

[ref11] Cunningham E. A. Stanovich E. K. (1997). Early reading acquisition and its relation to reading experience and ability 10 years later. Dev. Psychol. 33, 934–945. 9383616 10.1037//0012-1649.33.6.934

[ref12] Deary I. J. Penke L. Johnson W. (2010). The neuroscience of human intelligence differences. Nat. Rev. Neurosci. 11, 201–211. doi: 10.1038/nrn2793 20145623

[ref13] Demetriou A. Kazali E. Kazi S. Spanoudis G. (2020). Cognition and cognizance in preschool predict school achievement in primary school. Cogn. Dev. 54:100872. doi: 10.1016/j.cogdev.2020.100872

[ref14] Dolean D. D. Lervåg A. Visu-Petra L. Melby-Lervåg M. (2021). Language skills, and not executive functions, predict the development of reading comprehension of early readers: evidence from an orthographically transparent language. Read. Writ. 34:14911512. doi: 10.1007/s11145-020-10107-4

[ref15] Dumont R. Willis J. O. Elliott C. (2009). Essentials of DAS-II assessment. Hoboken, NJ: John Wiley & Sons.

[ref16] Dykiert D. Gale C. R. Deary I. J. (2009). Are apparent sex differences in mean IQ scores created in part by sample restriction and increased male variance? Intelligence 37, 42–47. doi: 10.1016/j.intell.2008.06.002

[ref17] Eliassen E. Zachrisson H. D. Melhuish E. (2017). Is cognitive development at three years of age associated with ECEC quality in Norway? Eur. Early Child. Educ. Res. J. 26, 97–110. doi: 10.1080/1350293X.2018.1412050

[ref18] Elliot C. D. (1983). The British ability scales. Windsor, England: NFER-Nelson.

[ref19] Elliot C. D. (1990a). DAS Administration and scoring manual. San Antonio, TX: The Psychological Corporation.

[ref20] Elliot C. D. (1990b). DAS introductory and technical handbook. San Antonio, TX: The Psychological Corporation.

[ref21] Elliot C. D. (2011). BAS3 – Administration and scoring manual. London: GL Assessment Limited.

[ref22] Elliot C. D. Smith P. (2011). British ability scales–third edition (BAS-3). London: GL Assessment Limited.

[ref23] Elliott C. D. (2001). “Application of the differential ability scales (DAS) and British ability scales, second edition (BAS II), for the assessment of learning disabilities” in Specific learning disabilities and difficulties in children and adolescents: Psychological assessment and evaluation. eds. Kaufman A. Kaufman N. (Cambridge Child and Adolescent Psychiatry), 178–217. Cambridge, England: Cambridge University Press.

[ref24] Elliott C. D. Smith P. McCulloch K. (1997). British ability scales second edition (BAS II). Technical manual. London: Nelson.

[ref25] Feingold A. (1994). Gender differences in variability in intellectual abilities: a cross-cultural perspective. Sex Roles 30, 81–92. doi: 10.1007/BF01420741

[ref26] Giofrè D. Allen K. Toffalini E. Caviola S. (2022). The impasse on gender differences in intelligence: a meta-analysis on WISC batteries. Educ. Psychol. Rev. 34, 2543–2568. doi: 10.1007/s10648-022-09705-1

[ref27] Gordon B. Elliott C. (2001). “Assessment with the differential ability scales” in Handbook of psychoeducational assessment. A practical handbook. eds. Phye G. D. Saklofske D. H. Andrews J. J. W. Janzen H. L. (Elsevier Science), 66–102. Cambridge MA, United States: Academic Press.

[ref28] Gottfredson L. (1997). Why g matters: the complexity of everyday life. Intelligence 24, 79–132.

[ref29] Hansen J. E. Broekhuizen M. L. (2021). Quality of the language learning environment and vocabulary development in early childhood. Scand. J. Educ. Res. 65, 302–317. doi: 10.1080/00313831.2019.1705894

[ref30] Hill V. (2005). Through the past darkly: a review of the British ability scales second edition. Child Adolesc. Mental Health 10, 87–98. doi: 10.1111/j.14753588.2004.00123.x 32806801

[ref31] Hirnstein M. Stuebs J. Moè A. Hausmann M. (2023). Sex/gender differences in verbal fluency and verbal-episodic memory: a meta-analysis. Perspect. Psychol. Sci. 18, 67–90. doi: 10.1177/17456916221082116 35867343 PMC9896545

[ref32] Holzhauer C. G. Cucciare M. Epstein E. E. (2020). Sex and gender effects in recovery from alcohol use disorder. Alcohol Res. 40:03. doi: 10.35946/arcr.v40.3.03 PMC7668196 33224697

[ref33] Horn J. L. McArdle J. J. (1992). A practical and theoretical guide to measurement invariance in aging research. Exp. Aging Res. 18, 117–144. doi: 10.1080/03610739208253916 1459160

[ref34] Hu L. Bentler P. M. (1999). Cutoff criteria for fit indexes in covariance structure analysis: conventional criteria versus new alternatives. Struct. Equ. Model. Multidiscip. J. 6, 1–55.

[ref35] IBM Corp. (2020). IBM SPSS statistics for windows, version 27.0. Armonk, NY: IBM Corp.

[ref36] Jäncke L. (2018). Sex/gender differences in cognition, neurophysiology, and neuroanatomy. F1000Res. 7:F1000 Faculty Rev-805. doi: 10.12688/f1000research.13917.1 PMC6013760 29983911

[ref37] Johnson W. Bouchard T. J. (2007). Sex differences in mental abilities: g masks the dimensions on which they lie. Intelligence 35, 23–39. doi: 10.1016/j.intell.2006.03.012

[ref38] Johnson J. L. Greaves L. Repta R. (2009). Better science with sex and gender: facilitating the use of a sex and gender-based analysis in health research. Int. J. Equity Health 8:14. doi: 10.1186/1475-9276-8-14 19419579 PMC2689237

[ref39] Johnson W. McGue M. Iacono W. G. (2006). Genetic and environmental influences on academic achievement trajectories during adolescence. Dev. Psychol. 42, 513–542. 10.1037/0012-1649.42.3.514 16756442

[ref40] Kievit R. A. Lindenberger U. Goodyer I. M. Jones P. B. Fonagy P. Bullmore E. T.. (2017). Mutualistic coupling between vocabulary and reasoning supports cognitive development during late adolescence and early adulthood. Psychol. Sci. 28, 1419–1431. doi: 10.1177/0956797617710785 28787239 PMC5641983

[ref41] Kline R. B., (2016). Principles and practice of structural equation modeling. 4th. New York: Guilford Press.

[ref42] Kotz K. M. Watkins M. W. McDermott P. A. (2008). Validity of the general conceptual ability score from the differential ability scales as a function of significant and rare interfactor variability. Sch. Psychol. Rev. 37, 261–278.

[ref43] Lövdén M. Fratiglioni L. Glymour M. M. Lindenberger U. Tucker-Drob E. M. (2020). Education and cognitive functioning across the life span. Psychol. Sci. Public Interest 21, 6–41. doi: 10.1177/1529100620920576 32772803 PMC7425377

[ref44] Mehmetoglu M. Jakobsen T. G., (2017). Applied statistics using STATA: a guide for the social sciences. London: SAGE Publications Ltd.

[ref45] Muthén L. K. Muthén B. O., (1998–2011). Mplus user's guide. 6th Los Angeles, CA: Muthén & Muthén.

[ref46] Muthén B. O. Muthén L. K. Asparouhov T., (2015). Estimator choices with categorical outcomes. Available at: https://www.statmodel.com/download/EstimatorChoices.pdf.

[ref47] OECD (2023). “Equity in education in PISA 2022” in PISA 2022 results (volume I): the state of learning and equity in education (Paris: OECD Publishing)

[ref48] Ortiz S. O. Oganes M. (2022). “Non-discriminatory, cross-cultural school neuropsychological assessment” in Best practices in school neuropsychology: guidelines for effective practice, assessment, and evidence-based intervention. eds. Miller D. C. Maricle D. E. Bedford C. L. Gettman J. A. (Hoboken, NJ: John Wiley & Sons), 41–64.

[ref49] Pace A. Luo R. Hirsh-Pasek K. Golinkoff R. M. (2017). Identifying pathways between socioeconomic status and language development. Annu. Rev. Linguist. 3, 285–308. doi: 10.1146/annurev-linguistics-011516-034226

[ref50] Palejwala M. H. Fine J. G. (2015). Gender differences in latent cognitive abilities in children aged 2 to 7. Intelligence 48, 96–108. doi: 10.1016/j.intell.2014.11.004

[ref51] Peng P. Kievit R. A. (2020). The development of academic achievement and cognitive abilities: a bidirectional perspective. Child Dev. Perspect. 14, 15–20. doi: 10.1111/cdep.12352 35909387 PMC7613190

[ref52] Price T. S. Eley T. C. Dale P. S. Stevenson J. Sandino K. Plomin R. (2000). Genetic and environmental covariation between verbal and non-verbal cognitive development in infancy. Child Dev. 71, 948–959. doi: 10.1111/1467-8624.00201, PMID: 11016558

[ref53] Raykov T. (2001). Bias of Cronbach’s coefficient alpha for fixed congeneric measures with correlated errors. Appl. Psychol. Meas. 25, 69–76.

[ref54] Reilly D. Neumann D. L. Andrews G. (2017). “Gender differences in spatial ability: implications for STEM education and approaches to reducing the gender gap for parents and educators” in Visual-spatial ability in STEM education. ed. Khine M. (Cham: Springer)

[ref55] Richardson K. Norgate S. H. (2015). Does IQ really predict job performance? Appl. Dev. Sci. 19, 153–169. doi: 10.1080/10888691.2014.983635 26405429 PMC4557354

[ref56] Romeo R. R. Flournoy J. C. McLaughlin K. A. Lengua L. J. (2022). Language development as a mechanism linking socioeconomic status to executive functioning development in preschool. Dev. Sci. 25:e13227. doi: 10.1111/desc.13227 34981872 PMC9250946

[ref57] Rowe M. L. Raudenbush S. W. Goldin-Meadow S. (2012). The pace of vocabulary growth helps predict later vocabulary skill. Child Dev. 83, 508–525. doi: 10.1111/j.1467-8624.2011.01710.x 22235920 PMC3262592

[ref58] Scarborough S. H. (2009). “Connecting early language and literacy to later Reading (dis)abilities: evidence, theory, and practice” in Approaching difficulties in literacy development assessment, pedagogy and Programmes. eds. Fletcher-Campbell F. Soler J. Reid G. (London, England: Sage). pp. 23–38.

[ref59] Sellers A. H. Burns W. J. Guyrke J. (2002). Differences in young Children's IQs on the Wechsler preschool and primary scale of intelligence-revised as a function of stratification variables. Appl. Neuropsychol. 9, 65–73. doi: 10.1207/S15324826AN0902_1 12214824

[ref60] Steinmayr R. Beauducel A. Spinath B. (2010). Do sex differences in a faceted model of fluid and crystallized intelligence depend on the method applied? Intelligence 38, 101–110. doi: 10.1016/j.intell.2009.08.001

[ref1001] Swinson J. (2013). British ability scales 3. Educ. Psychol. Pract. 29, 434–435. doi: 10.1080/02667363.2013.853380

[ref61] Tikhomirova T. Malykh A. Malykh S. (2020). Predicting academic achievement with cognitive abilities: cross-sectional study across school education. Behav. Sci. 10:158. doi: 10.3390/bs10100158 33080874 PMC7602962

[ref62] Van Iddekinge C. H. Aguinis H. Mackey J. D. DeOrtentiis P. S. (2018). A meta-analysis of the interactive, additive, and relative effects of cognitive ability and motivation on performance. J. Manag. 44, 249–279. doi: 10.1177/0149206317702220

[ref63] Wood W. Eagly A. H. (2012). “Biosocial construction of sex differences and similarities in behavior” in Advances in experimental social psychology. eds. Zanna M. P. Olso J. M., vol. 46 (Cambridge MA, United States: Academic Press), 55–123.

