# Supplementary material for: Verbal and non-verbal skills in early childhood: dimensionality, developmental trajectories, and gender differences
Source: Front Psychol. 2024 Apr 19;15:1330334. doi: 10.3389/fpsyg.2024.1330334 (PMC11066225; doi:10.3389/fpsyg.2024.1330334)
Supplement: Supplementary file 1 [file Table_1.DOCX]

# Supplementary Material

Verbal Expressive and Non-verbal Reasoning Skills in Early Childhood: Dimensionality, Developmental Trajectories, and Gender Differences

**Overview**

|  | **Appendix A. Distributions** |
| --- | --- |
| S1 | Distribution, Verbal Skills - Naming Vocabulary (T1) Mean Scores, Total Sample |
| S2 | Distribution, Verbal Skills - Naming Vocabulary (T2) Mean Scores, Total Sample |
| S3 | Distribution, Non-Verbal Skills – Picture Similarities (T1) Mean Scores, Total Sample |
| S4 | Distribution, Non-Verbal Skills – Picture Similarities (T2) Mean Scores, Total Sample |
|  | **Appendix B. Standardized item parameters** |
| S5 | Naming Vocabulary – CFA, Standardized item parameters, ages 3 and 5. |
| S6 | Picture Similarities – CFA, Standardized item parameters, ages 3 and 5. |
|  | **Appendix C. Correlations between latent factors** |
| S7 | Correlation between the Latent Factors, Total Sample. |
| S8 | Correlation between the Latent Factors, by Group: Girls / Boys. |
| S9 | Wald Test Results comparing Correlations across Gender |
|  | **Appendix D. About the Figures** |
| S10 | Individual CFA Results Including All Items. |
| S11 | Correlation between the Latent Factors, All Items, Total Sample. |
| S12 | Figure 3 |
| S13 | Figure 4 |
|  | **Appendix E. Correlations between the items** |
| S14 | Correlation Table, CFA, Naming Vocabulary – T1 (Age 3) with the Total Sample. |
| S15 | Correlation Table, CFA, Naming Vocabulary – T1 (Age 5) with the Total Sample. |
| S16 | Correlation Table, CFA, Picture Similarities – T1 (Age 3) with the Total Sample. |
| S17 | Correlation Table, Picture Similarities – T2 (Age 5) with the Total Sample. |

**Appendix A**

This is the distributions presented.

**S1. Distribution, Verbal Skills - Naming Vocabulary (T1) Mean Scores, Total Sample**


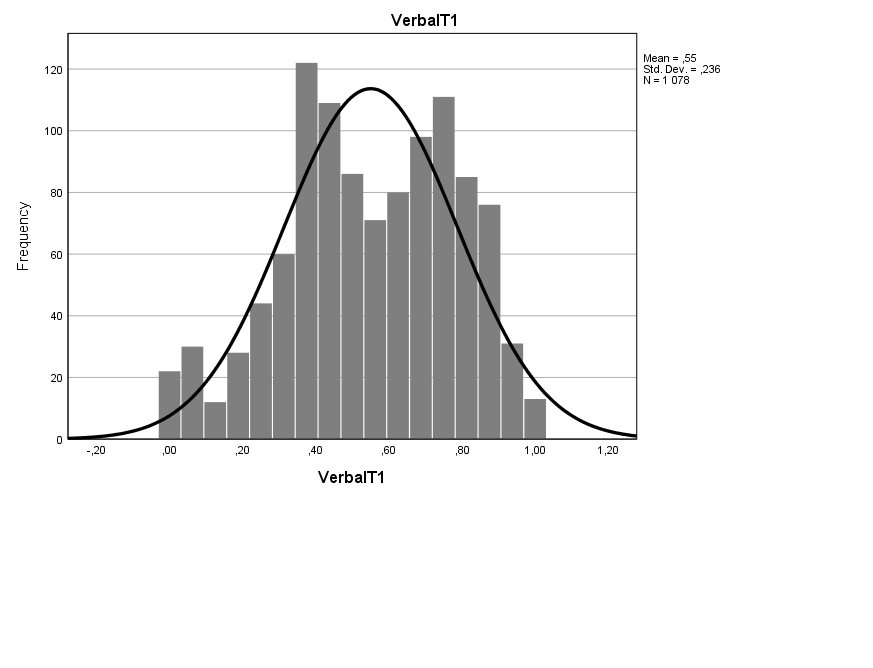

**S2. Distribution, Verbal Skills - Naming Vocabulary (T2) Mean Scores, Total Sample**


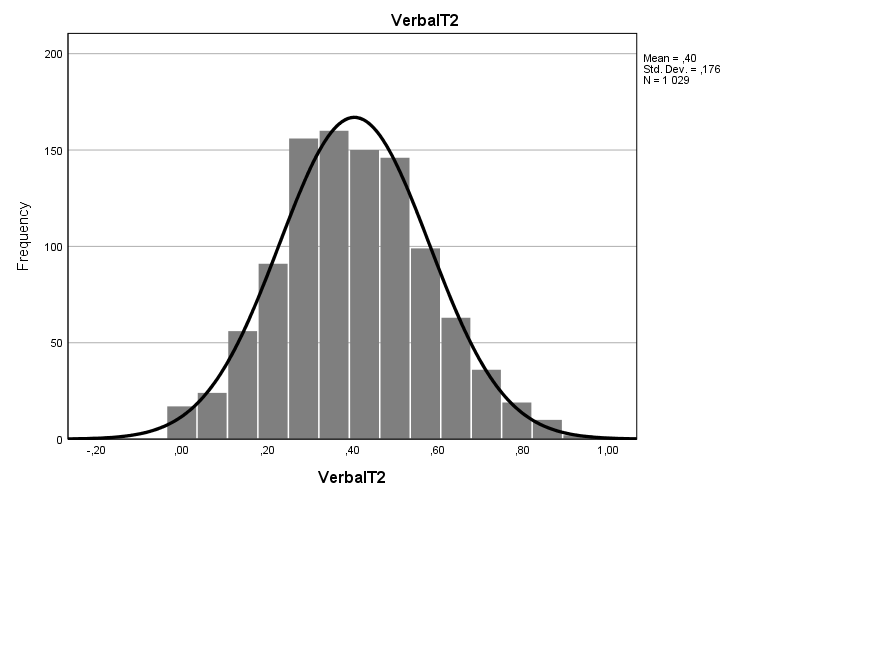


**S3. Distribution, Non-Verbal Skills – Picture Similarities (T1) Mean Scores, Total Sample**


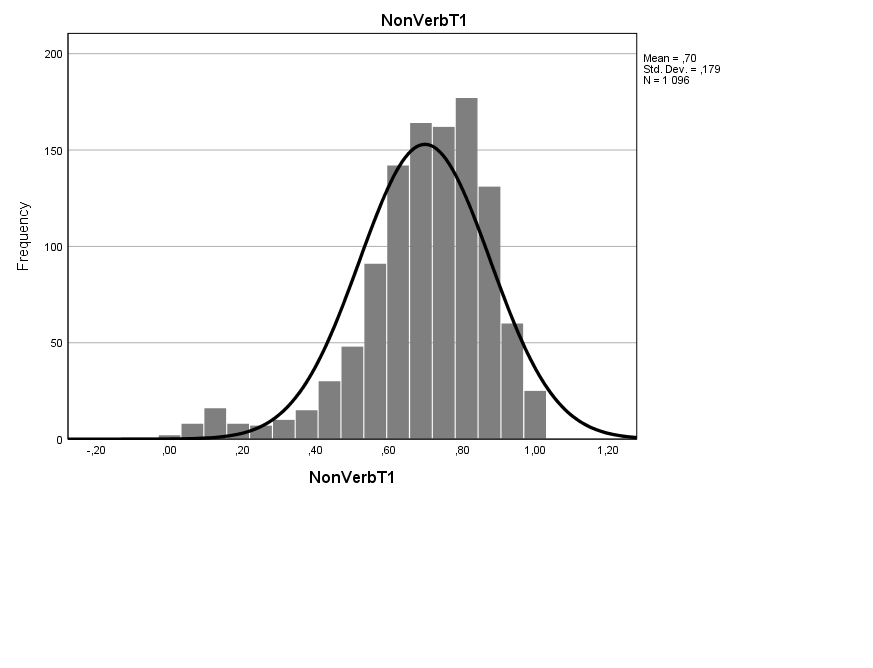

**S4. Distribution, Non-Verbal Skills – Picture Similarities (T2) Mean Scores, Total Sample**


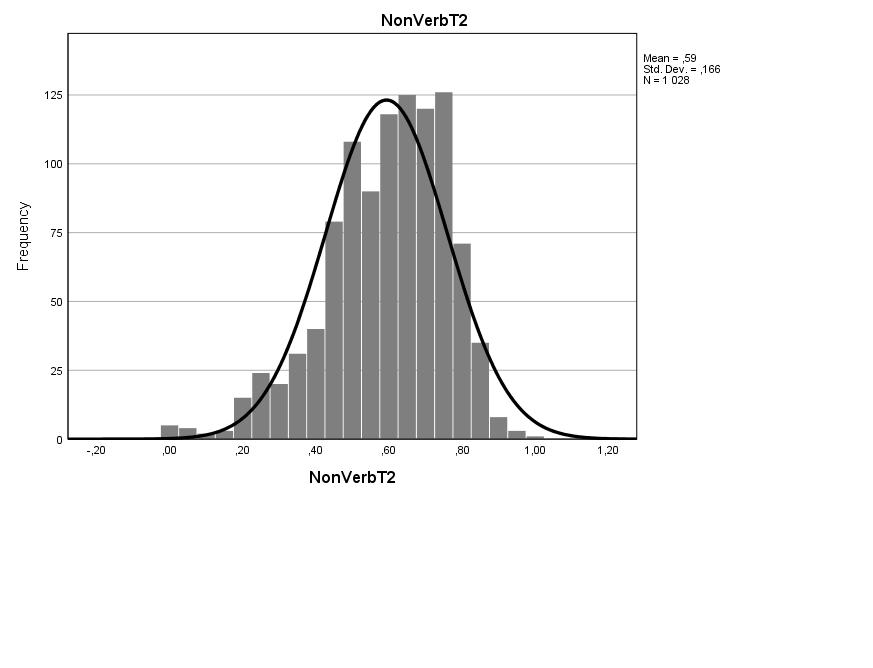


**Appendix B**
S5. Naming Vocabulary – CFA, Standardized item parameters, ages 3 and 5.

|  | Age 3 (N=1078) | | | Age 5 (N=1029) | | |
| --- | --- | --- | --- | --- | --- | --- |
| Variable | Correct (%) | Loadings | Thresholds | Correct (%) | Loadings | Thresholds |
| Item 5 | 89 | 0.467 | -1.200 |  |  |  |
| Item 8 | 86 | 0.728 | -1.059 |  |  |  |
| Item 9 | 51 | 0.463 | -0.033(ns) |  |  |  |
| Item 10 | 78 | 0.442 | -0.760 |  |  |  |
| Item 11 | 72 | 0.746 | -0.588 | NA | NA | NA |
| Item 12 | 66 | 0.593 | -0.414 | 79 | 0.229 | -0.814 |
| Item 14 | 86 | 0.797 | -1.088 | NA | NA | NA |
| Item 15 | 44 | 0.687 | 0.154 | NA | NA | NA |
| Item 16 | 39 | 0.549 | 0.278 | 84 | 0.634 | -0.977 |
| Item 17 | 83 | 0.739 | -0.959 | NA | NA | NA |
| Item 18 | 35 | 0.779 | 0.394 | NA | NA | NA |
| Item 19 | 33 | 0.777 | 0.434 | NA | NA | NA |
| Item 20 | 19 | 0.587 | 0.898 | 67 | 0.418 | -0.433 |
| Item 21 | 25 | 0.786 | 0.661 | 83 | 0.531 | -0.939 |
| Item 22 | 45 | 0.825 | 0.133 | NA | NA | NA |
| Item 23 | 30 | 0.795 | 0.517 | 73 | 0.579 | -0.603 |
| Item 24 | NA | NA | NA | 22 | 0.532 | 0.773 |
| Item 25 |  |  |  | 29 | 0.545 | 0.546 |
| Item 26 |  |  |  | 17 | 0.349 | 0.965 |
| Item 27 |  |  |  | 14 | 0.537 | 1.063 |
| Item 28 |  |  |  | 43 | 0.702 | 0.180 |
| Item 29 |  |  |  | 25 | 0.599 | 0.672 |
| Item 32 |  |  |  | 12 | 0.538 | 1.163 |
| Item 34 | |  |  | 11 | 0.622 | 1.233 |

*Note: ns. – not significant threshold.*

S6. Picture Similarities – CFA, Standardized item parameters, ages 3 and 5.

|  | T1 - Age 3 (N = 1096) | | | T2 - Age 5 (N = 1028) | | | |
| --- | --- | --- | --- | --- | --- | --- | --- |
| Indicator | Correct (%) | Loadings | Thresholds | Correct (%) | | Loadings | Thresholds |
| Item 3 | 85 | 0.320 | -1.030 |  | |  |  |
| Item 4 | 88 | 0.422 | -1.168 |  | |  |  |
| Item 5 | 68 | 0.356 | -0.459 |  | |  |  |
| Item 6 | 82 | 0.608 | -0.927 |  | |  |  |
| Item 7 | 86 | 0.385 | -1.058 |  | |  |  |
| Item 8 | 81 | 0.556 | -0.869 |  | |  |  |
| Item 9 | 76 | 0.551 | -0.695 |  | |  |  |
| Item 10 | 77 | 0.483 | -0.733 |  | |  |  |
| Item 11 | 68 | 0.470 | -0.475 |  | |  |  |
| Item 12 | 77 | 0.573 | -0.751 | NA | | NA | NA |
| Item 13 | 64 | 0.319 | -0.350 | 84 | | 0.284 | -1.013 |
| Item 14 | 55 | 0.445 | -0.124 | NA | | NA | NA |
| Item 15 | 48 | 0.425 | 0.048 (ns.) | NA | | NA | NA |
| Item 16 | 53 | 0.385 | -0.071 (ns.) | 88 | | 0.623 | -1.187 |
| Item 17 | 78 | 0.692 | -0.766 | NA | | NA | NA |
| Item 18 | 33 | 0.396 | 0.437 | 88 | | 0.584 | -1.172 |
| Item 19 |  |  |  | 87 | | 0.586 | -1.139 |
| Item 20 |  |  |  | 86 | | 0.426 | -1.063 |
| Item 21 |  |  |  | 79 | | 0.478 | -0.806 |
| Item 22 |  |  |  | 85 | | 0.495 | -1.046 |
| Item 23 |  |  |  | 73 | | 0.378 | -0.612 |
| Item 24 |  |  |  | 76 | | 0.476 | -0.690 |
| Item 25 |  |  |  | 64 | | 0.505 | -0.366 |
| Item 26 |  |  |  | 55 | | 0.416 | -0.115 |
| Item 27 |  |  |  | 55 | | 0.433 | -0.130 |
| Item 28 |  |  |  | 46 | | 0.430 | 0.103 |
| Item 29 |  |  |  | 46 | | 0.432 | 0.095 |
| Item 30 |  |  |  | 33 | | 0.484 | 0.441 |
| Item 31 |  |  |  | 32 | | 0.471 | 0.462 |
| Item 32 |  |  |  | 30 | | 0.449 | 0.517 |
| Item 33 |  |  |  | 31 | | 0.369 | 0.501 |
| Item 34 |  |  |  | 31 | 0.427 | | 0.495 |
| Item 35 |  |  |  | 17 | | 0.343 | 0.957 |

*Note: ns. – not significant threshold.*

**Appendix C**

S7. Correlation between the Latent Factors, Total Sample.

|  | Verbal T1 | Verbal T2 | Non-verbal T1 | Non-verbal T2 |
| --- | --- | --- | --- | --- |
| Verbal T1 | - |  |  |  |
| Verbal T2 | .661 | - |  |  |
| Non-verbal T1 | .413 | .242 | - |  |
| Non-verbal T2 | .235 | .377 | .268 | - |

*Note: Standardized results, with p-value < .00.*

S8. Correlation between the Latent Factors, by Group: Girls / Boys.

|  | Verbal T1 | Verbal T2 | Non-verbal T1 | Non-verbal T2 |
| --- | --- | --- | --- | --- |
| Verbal T1 | - |  |  |  |
| Verbal T2 | .676/.691 | - |  |  |
| Non-verbal T1 | .436/.395 | .366/.175* | - |  |
| Non-verbal T2 | .248/.230 | .450/.335 | .305/.234 | - |

*Note: Standardized results, with p-value < .00.*
** Significant group difference at p < .05.*

**S9. Wald Test Results comparing Correlations across Gender:**

Verbal T1 x Verbal T2: (1.036 (1) p = 0.3088).
Non-verbal T1 x Non-verbal T2: (0.816 (1), p = 0.3663).

Verbal T1 x Non-verbal T1: (0.286 (1) p = .5929).
Verbal T2 x Non-verbal T2: (1.147 (1) p = 0.2842).

Verbal T1 x Non-verbal T2: (0.059 (1), p = 0.8082).
Non-verbal T1 x Verbal T2: (4.275 (1), p = 0.0387).

**Appendix D**

S10. Individual CFA Results Including All Items.

| *All Items* | Naming Vocabulary/Verbal | Picture Similarities/Non-verbal |
| --- | --- | --- |
| T1 | χ^2^ = 533.298 (252), *p < .*000. RMSEA = .032 (CI = .028 - .036), CFI = .968, TLI = .965, WRMR = 1.343 | χ^2^ = 216.383 (135), *p < .*000. RMSEA = .023 (CI = .017 - .029), CFI = .947, TLI = .940, WRMR = 1.012 |
| Two-group | χ^2^ = 767.980 (526), *p < .*000. RMSEA = .029 (CI = .025 - .034), CFI = .969, TLI = .967, WRMR = 1.649 * | χ^2^ = 356.002 (286), *p < .*000. RMSEA = .021 (CI = .013 - .028), CFI = .953, TLI = .950, WRMR = 1.332 |
| T2 | χ^2^ = 455.957 (299), *p < .*000. RMSEA = .023 (CI = .018 - .027), CFI = .942, TLI = .937, WRMR = 1.388 | χ^2^ = 370.024 (252), *p < .*000. RMSEA = .021 (CI = .017 - .026), CFI = .941, TLI = .935, WRMR = 1.037 |
| Two-group | χ^2^ = 1043.090 (622), *p < .*000. RMSEA = .037 (CI = .033 - .040), CFI = .849, TLI = .842, WRMR = 2.321 * | χ^2^ = 606.262 (526), *p < .*000. RMSEA = .017 (CI = .009 - .023), CFI = .955, TLI = .953, WRMR = 1.345 |

**Note: Non-invariant across Gender.*

S11. Correlation between the Latent Factors, All Items, Total Sample.

|  | Verbal T1 | Verbal T2 | Non-verbal T1 | Non-verbal T2 |
| --- | --- | --- | --- | --- |
| Verbal T1 | - |  |  |  |
| Verbal T2 | .629 | - |  |  |
| Non-verbal T1 | .428 | .222 | - |  |
| Non-verbal T2 | .229 | .401 | .280 | - |

*Note: Standardized results, with p-value < .00.*

Model fit results: χ^2^ = 4620.443(4088), *p < .*000. RMSEA = .011 (CI = .009 - .012), CFI = .960, TLI = .959, WRMR = 1.095.

**S12. Figure 3**

SEM Model Step 1: χ^2^ = 4778.679(4180), *p < .*000. RMSEA = .011 (CI = .010 - .013), CFI = .948, TLI = .947, WRMR = 1.122.


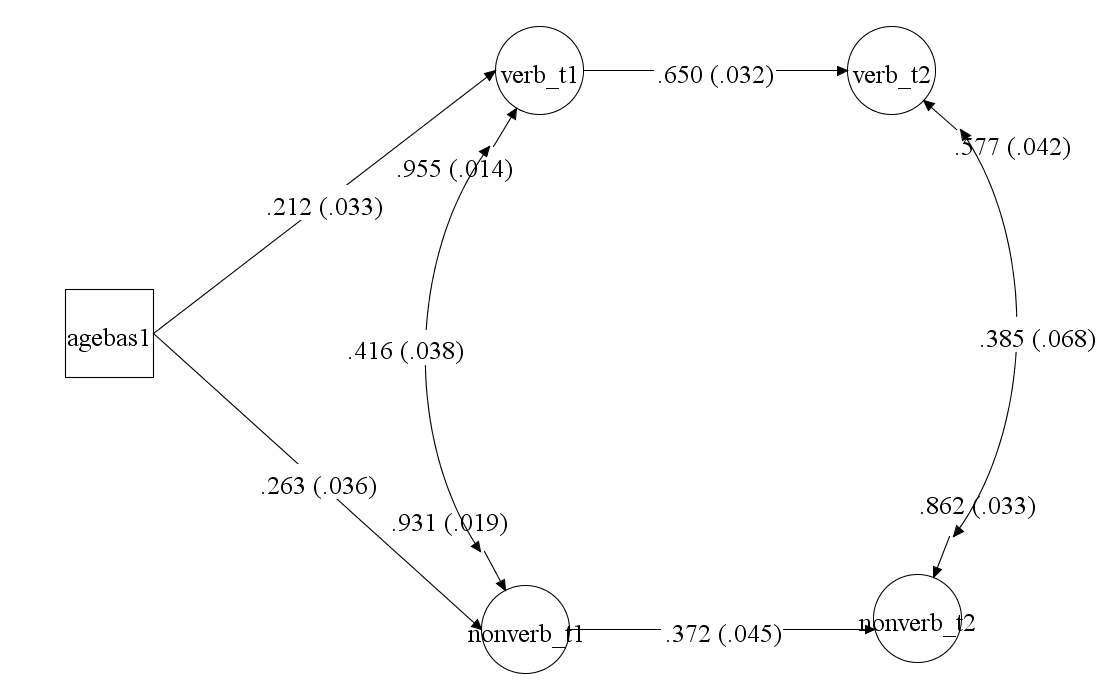


**S13. Figure 4**

SEM Model Step 1: χ^2^ = 4750.181(4178), *p < .*000. RMSEA = .011 (CI = .009 - .013), CFI = .950, TLI = .949, WRMR = 1.122.


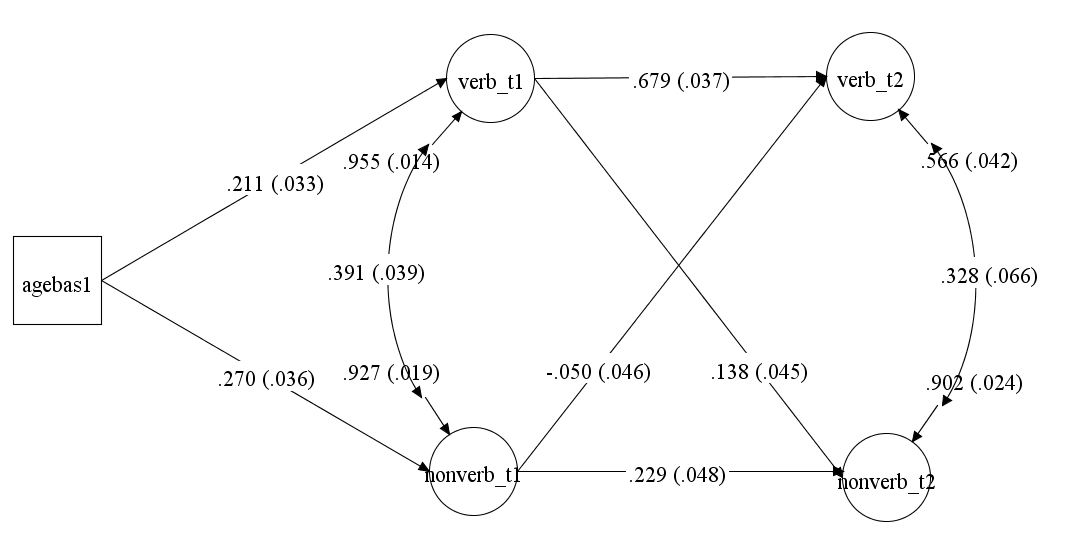


**Appendix E**

**S14. Correlation Table, CFA, Naming Vocabulary – T1 (Age 3) with the Total Sample.**

| Item | 5 | 8 | 9 | 10 | 11 | 12 | 14 | 15 | 16 | 17 | 18 | 19 | 20 | 21 | 22 | 23 |
| --- | --- | --- | --- | --- | --- | --- | --- | --- | --- | --- | --- | --- | --- | --- | --- | --- |
| 5 |  |  |  |  |  |  |  |  |  |  |  |  |  |  |  |  |
| 8 | 0.451 |  |  |  |  |  |  |  |  |  |  |  |  |  |  |  |
| 9 | 0.211 | 0.337 |  |  |  |  |  |  |  |  |  |  |  |  |  |  |
| 10 | 0.248 | 0.428 | 0.098 |  |  |  |  |  |  |  |  |  |  |  |  |  |
| 11 | 0.383 | 0.581 | 0.294 | 0.348 |  |  |  |  |  |  |  |  |  |  |  |  |
| 12 | 0.303 | 0.543 | 0.231 | 0.351 | 0.568 |  |  |  |  |  |  |  |  |  |  |  |
| 14 | 0.470 | 0.656 | 0.341 | 0.529 | 0.543 | 0.544 |  |  |  |  |  |  |  |  |  |  |
| 15 | 0.268 | 0.438 | 0.388 | 0.285 | 0.467 | 0.318 | 0.504 |  |  |  |  |  |  |  |  |  |
| 16 | 0.133 | 0.385 | 0.229 | 0.224 | 0.517 | 0.318 | 0.278 | 0.342 |  |  |  |  |  |  |  |  |
| 17 | 0.373 | 0.531 | 0.306 | 0.512 | 0.551 | 0.482 | 0.760 | 0.454 | 0.322 |  |  |  |  |  |  |  |
| 18 | 0.311 | 0.523 | 0.360 | 0.204 | 0.486 | 0.405 | 0.560 | 0.605 | 0.457 | 0.518 |  |  |  |  |  |  |
| 19 | 0.356 | 0.489 | 0.368 | 0.259 | 0.600 | 0.389 | 0.507 | 0.491 | 0.413 | 0.513 | 0.666 |  |  |  |  |  |
| 20 | 0.199 | 0.424 | 0.305 | 0.263 | 0.458 | 0.276 | 0.411 | 0.528 | 0.298 | 0.376 | 0.456 | 0.363 |  |  |  |  |
| 21 | 0.362 | 0.526 | 0.342 | 0.143 | 0.580 | 0.355 | 0.489 | 0.624 | 0.393 | 0.419 | 0.670 | 0.636 | 0.472 |  |  |  |
| 22 | 0.345 | 0.484 | 0.405 | 0.355 | 0.515 | 0.500 | 0.565 | 0.536 | 0.529 | 0.538 | 0.625 | 0.698 | 0.499 | 0.700 |  |  |
| 23 | 0.314 | 0.542 | 0.436 | 0.202 | 0.626 | 0.387 | 0.488 | 0.544 | 0.484 | 0.504 | 0.629 | 0.649 | 0.468 | 0.632 | 0.701 |  |

**S15. Correlation Table, CFA, Naming Vocabulary – T2 (Age 5) with the Total Sample.**

| Item | 12 | 16 | 20 | 21 | 23 | 24 | 25 | 26 | 27 | 28 | 29 | 32 | 34 |
| --- | --- | --- | --- | --- | --- | --- | --- | --- | --- | --- | --- | --- | --- |
| 12 |  |  |  |  |  |  |  |  |  |  |  |  |  |
| 16 | 0.270 |  |  |  |  |  |  |  |  |  |  |  |  |
| 20 | 0.045 | 0.283 |  |  |  |  |  |  |  |  |  |  |  |
| 21 | 0.272 | 0.496 | 0.280 |  |  |  |  |  |  |  |  |  |  |
| 23 | 0.153 | 0.330 | 0.260 | 0.432 |  |  |  |  |  |  |  |  |  |
| 24 | 0.132 | 0.377 | 0.236 | 0.259 | 0.237 |  |  |  |  |  |  |  |  |
| 25 | 0.076 | 0.278 | 0.272 | 0.214 | 0.305 | 0.343 |  |  |  |  |  |  |  |
| 26 | 0.010 | 0.175 | 0.078 | 0.093 | 0.252 | 0.369 | 0.220 |  |  |  |  |  |  |
| 27 | 0.090 | 0.348 | 0.062 | 0.243 | 0.292 | 0.243 | 0.316 | 0.102 |  |  |  |  |  |
| 28 | 0.163 | 0.411 | 0.223 | 0.253 | 0.438 | 0.294 | 0.401 | 0.235 | 0.427 |  |  |  |  |
| 29 | 0.050 | 0.293 | 0.295 | 0.332 | 0.287 | 0.324 | 0.343 | 0.225 | 0.313 | 0.482 |  |  |  |
| 32 | 0.055 | 0.268 | 0.300 | 0.162 | 0.280 | 0.212 | 0.283 | 0.107 | 0.372 | 0.459 | 0.289 |  |  |
| 34 | 0.101 | 0.406 | 0.285 | 0.119 | 0.315 | 0.391 | 0.306 | 0.225 | 0.410 | 0.437 | 0.368 | 0.399 |  |

**S16. Correlation Table, CFA, Picture Similarities – T1 (Age 3) with the Total Sample.**

| Item | 3 | 4 | 5 | 6 | 7 | 8 | 9 | 10 | 11 | 12 | 13 | 14 | 15 | 16 | 17 | 18 |
| --- | --- | --- | --- | --- | --- | --- | --- | --- | --- | --- | --- | --- | --- | --- | --- | --- |
| 3 |  |  |  |  |  |  |  |  |  |  |  |  |  |  |  |  |
| 4 | 0.292 |  |  |  |  |  |  |  |  |  |  |  |  |  |  |  |
| 5 | 0.079 | 0.336 |  |  |  |  |  |  |  |  |  |  |  |  |  |  |
| 6 | 0.318 | 0.343 | 0.127 |  |  |  |  |  |  |  |  |  |  |  |  |  |
| 7 | 0.065 | 0.014 | 0.062 | 0.169 |  |  |  |  |  |  |  |  |  |  |  |  |
| 8 | 0.208 | 0.159 | 0.262 | 0.392 | 0.277 |  |  |  |  |  |  |  |  |  |  |  |
| 9 | 0.159 | 0.244 | 0.269 | 0.426 | 0.270 | 0.243 |  |  |  |  |  |  |  |  |  |  |
| 10 | 0.207 | 0.181 | 0.162 | 0.264 | 0.162 | 0.244 | 0.234 |  |  |  |  |  |  |  |  |  |
| 11 | 0.140 | 0.179 | 0.131 | 0.358 | 0.198 | 0.232 | 0.303 | 0.296 |  |  |  |  |  |  |  |  |
| 12 | 0.175 | 0.243 | 0.287 | 0.277 | 0.209 | 0.330 | 0.241 | 0.371 | 0.329 |  |  |  |  |  |  |  |
| 13 | 0.018 | 0.091 | 0.084 | 0.230 | 0.118 | 0.210 | 0.152 | 0.093 | 0.145 | 0.196 |  |  |  |  |  |  |
| 14 | 0.148 | 0.237 | 0.149 | 0.232 | 0.144 | 0.174 | 0.265 | 0.227 | 0.161 | 0.146 | 0.180 |  |  |  |  |  |
| 15 | 0.067 | 0.148 | 0.129 | 0.242 | 0.217 | 0.281 | 0.273 | 0.136 | 0.169 | 0.185 | 0.124 | 0.255 |  |  |  |  |
| 16 | 0.056 | 0.116 | 0.077 | 0.171 | 0.239 | 0.203 | 0.203 | 0.106 | 0.127 | 0.234 | 0.154 | 0.274 | 0.215 |  |  |  |
| 17 | 0.241 | 0.253 | 0.186 | 0.372 | 0.275 | 0.403 | 0.329 | 0.359 | 0.314 | 0.429 | 0.287 | 0.307 | 0.284 | 0.277 |  |  |
| 18 | 0.000 | 0.064 | 0.144 | 0.205 | 0.157 | 0.180 | 0.245 | 0.232 | 0.102 | 0.245 | 0.053 | 0.226 | 0.203 | 0.210 | 0.345 |  |

**S17. Correlation Table, Picture Similarities – T2 (Age 5) with the Total Sample.**

|  | 13 | | 16 | 18 | 19 | 20 | 21 | 22 | 23 | 24 | 25 | 26 | 27 | 28 | 29 | 30 | 31 | 32 | 33 | 34 | 35 |
| --- | --- | --- | --- | --- | --- | --- | --- | --- | --- | --- | --- | --- | --- | --- | --- | --- | --- | --- | --- | --- | --- |
| 13 | |  |  |  |  |  |  |  |  |  |  |  |  |  |  |  |  |  |  |  |  |
| 16 | | 0.299 |  |  |  |  |  |  |  |  |  |  |  |  |  |  |  |  |  |  |  |
| 18 | | 0.211 | 0.434 |  |  |  |  |  |  |  |  |  |  |  |  |  |  |  |  |  |  |
| 19 | | 0.154 | 0.304 | 0.341 |  |  |  |  |  |  |  |  |  |  |  |  |  |  |  |  |  |
| 20 | | 0.017 | 0.203 | 0.291 | 0.341 |  |  |  |  |  |  |  |  |  |  |  |  |  |  |  |  |
| 21 | | 0.201 | 0.407 | 0.497 | 0.329 | 0.216 |  |  |  |  |  |  |  |  |  |  |  |  |  |  |  |
| 22 | | 0.090 | 0.275 | 0.199 | 0.357 | 0.316 | 0.227 |  |  |  |  |  |  |  |  |  |  |  |  |  |  |
| 23 | | 0.045 | 0.199 | 0.248 | 0.142 | 0.240 | 0.066 | 0.246 |  |  |  |  |  |  |  |  |  |  |  |  |  |
| 24 | | 0.180 | 0.207 | 0.297 | 0.315 | 0.176 | 0.135 | 0.276 | 0.213 |  |  |  |  |  |  |  |  |  |  |  |  |
| 25 | | 0.116 | 0.308 | 0.220 | 0.297 | 0.201 | 0.259 | 0.296 | 0.160 | 0.279 |  |  |  |  |  |  |  |  |  |  |  |
| 26 | | 0.065 | 0.209 | 0.190 | 0.284 | 0.222 | 0.134 | 0.241 | 0.152 | 0.220 | 0.236 |  |  |  |  |  |  |  |  |  |  |
| 27 | | 0.145 | 0.327 | 0.210 | 0.281 | 0.157 | 0.244 | 0.167 | 0.186 | 0.163 | 0.294 | 0.146 |  |  |  |  |  |  |  |  |  |
| 28 | | 0.087 | 0.314 | 0.261 | 0.278 | 0.174 | 0.233 | 0.137 | 0.257 | 0.193 | 0.200 | 0.230 | 0.146 |  |  |  |  |  |  |  |  |
| 29 | | 0.082 | 0.278 | 0.265 | 0.233 | 0.190 | 0.126 | 0.217 | 0.137 | 0.214 | 0.265 | 0.128 | 0.311 | 0.061 |  |  |  |  |  |  |  |
| 30 | | 0.148 | 0.280 | 0.274 | 0.207 | 0.183 | 0.185 | 0.289 | 0.257 | 0.249 | 0.190 | 0.230 | 0.150 | 0.371 | 0.097 |  |  |  |  |  |  |
| 31 | | 0.123 | 0.268 | 0.169 | 0.180 | 0.238 | 0.180 | 0.237 | 0.104 | 0.133 | 0.260 | 0.215 | 0.168 | 0.210 | 0.269 | 0.280 |  |  |  |  |  |
| 32 | | 0.176 | 0.346 | 0.233 | 0.224 | 0.138 | 0.237 | 0.191 | 0.183 | 0.281 | 0.221 | 0.220 | 0.173 | 0.141 | 0.100 | 0.154 | 0.261 |  |  |  |  |
| 33 | | 0.063 | 0.121 | 0.148 | 0.144 | 0.096 | 0.064 | 0.174 | 0.125 | 0.207 | 0.121 | 0.154 | 0.169 | 0.086 | 0.304 | 0.240 | 0.216 | 0.193 |  |  |  |
| 34 | | 0.068 | 0.219 | 0.230 | 0.352 | 0.126 | 0.120 | 0.064 | 0.184 | 0.184 | 0.242 | 0.197 | 0.150 | 0.152 | 0.220 | 0.153 | 0.245 | 0.201 | 0.308 |  |  |
| 35 | | 0.198 | 0.237 | 0.080 | 0.166 | 0.035 | 0.062 | 0.220 | 0.097 | 0.206 | 0.075 | 0.069 | 0.108 | 0.088 | 0.208 | 0.220 | 0.254 | 0.253 | 0.152 | 0.236 |  |
